# Stalling the Course of Neurodegenerative Diseases: Could Cyanobacteria Constitute a New Approach toward Therapy?

**DOI:** 10.3390/biom13101444

**Published:** 2023-09-25

**Authors:** Vitória Ramos, Mariana Reis, Leonor Ferreira, Ana Margarida Silva, Ricardo Ferraz, Mónica Vieira, Vitor Vasconcelos, Rosário Martins

**Affiliations:** 1 School of Health, Polytechnic Institute of Porto (ESS/P.PORTO), Rua Dr. António Bernardino de Almeida 400, 4200-072 Porto, Portugal; mvitorianetor@gmail.com (V.R.); agl@ess.ipp.pt (A.M.S.); rferraz@ess.ipp.pt (R.F.); mav@ess.ipp.pt (M.V.); 2 Interdisciplinary Centre of Marine and Environmental Research, University of Porto (CIIMAR/CIMAR), Terminal de Cruzeiros do Porto de Leixões, Av. General Norton de Matos s/n, 4450-208 Matosinhos, Portugal; mreis@ciimar.up.pt (M.R.); lferreira@ciimar.up.pt (L.F.); vmvascon@fc.up.pt (V.V.); 3 Department of Biology, Faculty of Sciences, University of Porto (FCUP), Rua do Campo Alegre, Edifício FC4, 4169-007 Porto, Portugal; 4 Associated Laboratory for Green Chemistry—Network of Chemistry and Technology (LAQV-REQUIMTE), Departamento de Química e Bioquímica, Faculdade de Ciências, Universidade do Porto, Rua do Campo Alegre 687, 4169-007 Porto, Portugal; 5 Center for Translational Health and Medical Biotechnology Research (TBIO/ESS/P.PORTO), Rua Dr. António Bernardino de Almeida 400, 4200-072 Porto, Portugal

**Keywords:** cyanobacteria, spirulina, neurodegenerative diseases, natural products

## Abstract

Neurodegenerative diseases (NDs) are characterized by progressive and irreversible neuronal loss, accompanied by a range of pathological pathways, including aberrant protein aggregation, altered energy metabolism, excitotoxicity, inflammation, and oxidative stress. Some of the most common NDs include Alzheimer’s Disease (AD), Parkinson’s Disease (PD), Multiple Sclerosis (MS), Amyotrophic Lateral Sclerosis (ALS), and Huntington’s Disease (HD). There are currently no available cures; there are only therapeutic approaches that ameliorate the progression of symptoms, which makes the search for new drugs and therapeutic targets a constant battle. Cyanobacteria are ancient prokaryotic oxygenic phototrophs whose long evolutionary history has resulted in the production of a plethora of biomedically relevant compounds with anti-inflammatory, antioxidant, immunomodulatory, and neuroprotective properties, that can be valuable in this field. This review summarizes the major NDs and their pathophysiology, with a focus on the anti-neurodegenerative properties of cyanobacterial compounds and their main effects.

## 1. Introduction

Neurodegenerative diseases (NDs) are a broad category of neurological ailments that induce progressive and irreversible neuronal loss in the central and peripheral nervous system (CNS and PNS, respectively) [1]. The loss of neurons, which are unable to efficiently regenerate owing to their terminally differentiated nature, promotes the collapse of functional neuronal networks and the loss of synaptic plasticity, impairing brain and nerve function. This results in a wide and often overlapping spectrum of symptoms typical of these disorders, such as impaired memory, cognition, behavior, sensory, and/or motor function [2].

Common NDs include Alzheimer’s Disease (AD), Parkinson’s Disease (PD), Multiple Sclerosis (MS), Amyotrophic Lateral Sclerosis (ALS), and Huntington’s Disease (HD). Each disease differs in clinical presentation and underlying physiology but they all share converging neurodegenerative pathways that lead to neuronal death, such as aberrant protein aggregation, neuroinflammation, oxidative stress, altered energy metabolism, and excitotoxicity [1]. Although the etiology of these diseases is multifactorial, aging is the primary risk factor because it is a natural process involving the dysregulation of multiple pathways implicated in neurodegeneration. However, environmental factors, genetic makeup, and other medical disorders, such as metabolic diseases, can all play a role [3].

NDs place significant health, social, and economic burdens on patients and caregivers and represent a serious public health concern. Millions of individuals are affected worldwide and this number is predicted to escalate rapidly as the population and life expectancy increase, making it a leading cause of mortality and morbidity [4].

NDs are complex diseases with multiple factors involved in their origin and progression. Despite extensive research, most attempts to develop effective treatments have been unsuccessful, many due to adverse side effects such as nausea, diarrhea, fatigue, hepatotoxicity, bradycardia, and secondary autoimmune adverse effects [2,5]. Currently, there are no therapeutic options to reverse the onset of NDs. Most of the few approved drugs such as the acetylcholinesterase (AChE) inhibitors donepezil, rivastigmine, and galantamine, and the N-methyl-D-aspartate (NMDA) receptor antagonist memantine for AD [6]; dopaminergic drugs such as levodopa for PD [7]; riluzole and edaravone for ALS [8]; and tetrabenazine and deutetrabenazine to reduce chorea in HA [9] only provide symptom management, while disease-modifying drugs are still in their infancy. Therefore, most conditions progress without remission and are ultimately fatal. Given the gravity and rising prevalence of NDs, it is imperative to identify new and effective pharmacological candidates and targets [5].

Although compounds produced naturally by our body are considered promising in the treatment of NDs, such as melatonin and the immunosuppressive cytokine IL-10 [10,11], natural products derived from plants, algae, macrofungi, invertebrates, and microorganisms have traditionally been key contributors to drug development due to their great diversity and structural complexity [12]. Natural compounds, synthetic derivatives, and pharmacophore-inspired drugs account for more than 60% of all approved drugs [13].

Cyanobacteria are primitive prokaryotes that produce several bioactive metabolites with diverse pharmacological properties, such as being neuroprotective, antioxidant, anti-inflammatory, and immunomodulatory [14,15,16], which can be an asset in the treatment of NDs.

Given the ubiquity of NDs and the potential of cyanobacteria in innovative treatment options, the purpose of this review is to compile existing evidence on the potential of cyanobacteria-derived products to combat neurodegeneration and the major NDs.

## 2. Cyanobacteria

Cyanobacteria, also known as green–blue algae, are a diverse phylum of gram-negative microorganisms that are unique in their ability to perform oxygenic photosynthesis, setting them apart from other prokaryotes [17]. Cyanobacteria were among the first species to live on Earth, with more than 3.5 billion years of fossil records. These organisms are key oxygen producers and nitrogen fixers that play important roles in ecosystems and in shaping the biosphere [18]. Cyanobacteria exhibit diverse morphologies, ranging from single cells to colonies and filaments, and can be present at high densities, such as in crusts or blooms. They thrive in a wide range of environments, including freshwater, marine, and terrestrial ecosystems, even those deemed hostile to life [17]. Their ability to adapt and survive is a result of their metabolic diversity, flexibility, and reactivity, which involves unique biochemical pathways that yield a variety of metabolites including proteins, essential fatty acids, vitamins, minerals, flavonoids, carotenoids, chlorophylls, and phycobiliproteins [18,19]. Cyanobacteria also offer economic and sustainable advantages as they have a fast-growing potential with high yields without the need for many resources, making them an appealing option for biomedical research [20].

Cyanobacteria’s health benefits have long been documented as *Nostoc* species have been used to treat gout, fistulas, and cancer since 1500 B.C. and as Aztecs employed *Spirulina* strains as a food source [20,21]. *Spirulina* remains one of the most extensively studied genera of cyanobacteria and is widely used as a dietary supplement due to its impressive health benefits and nutritional makeup, which includes a high protein content (60–70% of dry weight), vitamin B12, essential fatty acids, polysaccharides, and various pigments such as β-carotene and phycocyanin, one of the most biologically active components [20].

Several cyanobacteria-derived metabolites have been identified, exhibiting anti-cancer, anti-viral, anti-bacterial, and anti-diabetic properties, among others. Some of these, such as the anticancer drug Adcetris^TM^, are in commercial use, whereas others are undergoing preclinical and clinical trials [20,22,23].

Regarding neuroprotection, cyanobacteria produce several neuroactive compounds that have been linked to ecological roles, such as enhancing competitiveness in grazing defenses by reducing palatability and repelling predators [24]. However, the effects of cyanobacteria-derived products can vary widely, from the medicinal potential of phycocyanin to lethal cyanotoxins like microcystins, nodularin, and β-N-methylamino-L-alanine (BMAA), whose exposure has been associated with the onset of NDs [24,25].

## 3. Neurodegeneration

Neurodegeneration is a complex process characterized by the progressive structural and functional loss of neuronal cells in the CNS and PNS; it is the primary pathologic feature of NDs. Several pathways, including abnormal protein aggregation, oxidative stress, neuroinflammation, excitotoxicity, mitochondrial dysfunction, and apoptosis, have been implicated in the pathogenesis of neurodegeneration [1]. In this context, cyanobacterial compounds exhibit a variety of properties that can aid in the battle against neurodegenerative processes. This section provides a brief overview of the key hallmarks of neurodegeneration and how cyanobacterial natural products can help ameliorate them.

Pathological protein aggregation is a typical trait of NDs and contributes to their diagnosis and categorization. Many NDs are proteinopathies caused by the abnormal aggregation of proteins, such as β-amyloid (Aβ) and tau in AD, α-synuclein in PD, or TAR DNA-binding protein 43 (TDP-43) in ALS [26]. Protein misfolding and oligomerization lead to extracellular or intracellular aggregates, which can appear as oligomers, amorphous assemblies, or highly structured amyloid fibrils and plaques. This is often favored by gene mutations, post-translational modifications, or inadequate proteostasis and protein quality control [27]. Protein aggregates spread in a prion-like manner, with a protein seed enlisting normally folded molecules to adopt abnormal conformations [28]. Aggregate toxicity is mostly mediated by gain-of-function, resulting in cellular dysfunction, synaptic loss, and brain injury [26,27]. Cyanobacterial natural products have shown the potential to alleviate proteotoxicity. For example, the patented Klamin^®^ extract from *Aphanizomenon flos-aquae*, rich in phenylethylamine, interferes with Aβ aggregation kinetics on a cellular model [29] and phycocyanin from *Leptolyngbya* sp. N62DM reduces the polyglutamine (polyQ) aggregation in a worm model of HD [30].

Most NDs are also linked to elevated levels of oxidative stress markers. Oxidative stress is caused by an imbalance between the production of reactive oxygen and nitrogen species (ROS and RNS) and the antioxidant defense system. The CNS is particularly vulnerable to oxidative stress because of its high metabolic rate and oxidizable substrate content [31]. A pro-oxidant state promotes lipid, protein, and DNA damage as well as cellular injury and mitochondrial malfunction, all of which contribute to neurodegeneration. In a complex and reciprocal interplay, oxidative stress promotes many traditional neurodegenerative pathways while also being aggravated by events such as aberrant protein aggregation and metal homeostasis loss [32]. There is substantial evidence that *Spirulina* and other cyanobacteria have strong antioxidant capacity, enhancing the antioxidant defense system, scavenging ROS, inhibiting lipid peroxidation, and modulating genes related to the oxidative stress response [33,34,35,36,37].

Another common feature in NDs is chronic neuroinflammation. The inflammatory response in the brain is mediated by microglia and astrocytes. Harmful *stimuli*, such as protein aggregation and oxidative stress, activate glial cells causing their phenotype to shift from neuroprotective to pro-inflammatory. While decreasing their phagocytic function, activated microglia release pro-inflammatory mediators such as tumor necrosis factor (TNF)-α, interleukin (IL)-1β, IL-16, nitric oxide (NO), and chemokines [38]. These mediators stimulate astrocytes to activate further reactions that can impair synaptic function, the blood–brain barrier, metabolic function, and glutamate metabolism, further exacerbating neurodegeneration [38,39]. Cyanobacteria possess strong anti-inflammatory properties that have been shown to impact microglial activation and response, decrease inflammatory mediators, and modulate inflammatory genes [40,41,42,43,44].

**Figure 1:**
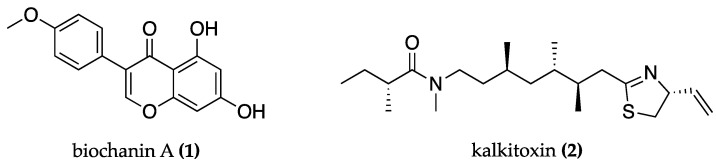
Structure of biochanin-A **(1)**, a phytoestrogen that prevents mitochondria dysfunction, and kalkitoxin **(2)**, a lipopeptide that interacts with voltage-sensitive sodium channels.

Excitotoxicity is an abnormal process of neuronal death caused by pathologically high levels of excitatory neurotransmitters, primarily glutamate. This amplifies or prolongs the activation of glutamate receptors, causing rapid and prolonged calcium (Ca^2+^) influx into neurons, which triggers several Ca^2+^-dependent enzymes that initiate a neurotoxic cascade [45]. This has negative implications such as mitochondrial malfunction, ROS overproduction, and the release of pro-apoptotic proteins, among others. Mitochondria are particularly sensitive because they capture excess cytosolic Ca^2+^, causing the mitochondrial permeability transition pore to open, resulting in energy malfunction and the activation of apoptotic cell death pathways [45]. In this context, cyanobacteria-derived products have shown promise. For instance, phycocyanin from *Spirulina* sp. inhibits cellular glutamate excitotoxicity [46]; biochanin A **(1)** (Figure 1), which has been identified in cyanobacterial blooms, prevents mitochondrial dysfunction and related cellular apoptosis [47,48] and kalkitoxin **(2)** (Figure 1) from *Lyngbya majuscula* inhibits the elevation of Ca^2+^ in neurons as it is a voltage-gated ion channel inhibitor [49].

Neurodegeneration is a complex process and different pathological pathways may play varying roles in the development of each ND. Since these processes are intertwined, addressing many modes of action through combinatorial multi-target therapy, such as the use of cyanobacteria, is a promising strategy for ND prevention and treatment [1].

## 4. Cyanobacteria Potential against Neurodegenerative Diseases

There are numerous examples in the literature of cyanobacteria’s potential as a source of compounds or extracts with potential in ND therapy. The following section reviews the main characteristics of the major NDs and the therapeutic potential of cyanobacteria-derived compounds or extracts.

### 4.1. Cyanobacteria against Alzheimer’s Disease

Alzheimer’s Disease (AD) is an age-related ND that mostly affects patients aged 65 years and older [6]. It is the leading cause of dementia, accounting for 60–70% of the estimated 50 million total cases [50]. It is characterized by two main neuropathological features in the brain: the extracellular deposition of senile plaques composed of Aβ-peptide and the accumulation of intracellular hyperphosphorylated tau protein in neurofibrillary tangles. These, along with other pathological processes such as acetylcholine deficiency, vascular damage, oxidative stress, inflammation, and mitochondrial dysfunction, lead to neuronal death and atrophy, primarily in the entorhinal cortex and hippocampus, resulting in severe cognitive impairment, memory loss, and behavioral changes [51]. AD can have multiple causes, such as genetic mutations, mainly in the amyloid precursor protein (APP), presenilin-1 (PSEN-1), presenilin-2 (PSEN-2), and apolipoprotein E (ApoE) genes; lifestyle and environmental factors; and other medical issues [6].

The two main pharmacological classes used in AD are AChE inhibitors, namely donepezil, rivastigmine, and galantamine, and the NMDA receptor antagonist memantine. However, these options only provide temporary symptom relief, failing to halt or regress the progression of the disease [52]. Other potential treatment targets can include immunotherapy, small-molecule inhibitors, antioxidants, and anti-inflammatory drugs [52,53].

The potential of cyanobacteria against AD is vast, as reviewed by Castaneda et al. (2021) [24]. Recent studies have reinforced this hypothesis (Table 1).

One of the most explored treatment approaches for AD is restoring cholinergic signaling. In AD patients, low levels of the neurotransmitters acetylcholine (ACh) and butyrylcholine (BCh) and high expression of AChE and butyrylcholinesterase (BChE) were reported [54]. The strategy of inhibiting these enzymes, which hydrolyze ACh and BCh, increases their concentration in the synaptic cleft and thus reduces symptoms [54].

**Figure 2:**
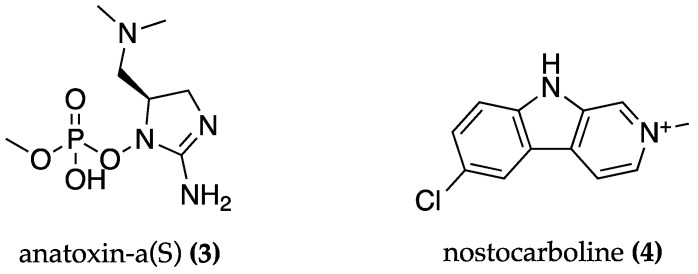
Structure of anatoxin-a(S) **(3)** and nostocarboline **(4)**, cyanobacteria-derived AChE and BChE inhibitors.

Cyanobacteria-derived AChE and BChE inhibitors were reported. Anatoxin-a(S) **(3)** (Figure 2) from *Anabaena flos-aquae* is an irreversible AChE inhibitor but it is also a potent neurotoxin that can cause severe cholinergic poisoning when administered to rats (0.1–1.0 mg/kg) [55]. Nostocarboline **(4)** (Figure 2) from *Nostoc* is an inhibitor of AChE and BChE, with half-maximal inhibitory concentration (IC_50_) values of 5.3 μM [56] and 13.2 µM [57], respectively. However, it is also a neurotoxin, showing moderate toxicity when tested in crustaceans [56]. Although described as a potent neurotoxin produced by cyanobacteria, anatoxin-a(S) is also one of the least understood and monitored [58]. In fact, as recently reviewed, studies involving cyanobacteria neurotoxins such as anatoxin-a (S) in standardized neuronal cell lines and mammals are still scarce and results are inadequate to confirm its real toxicity [59].

**Figure 3:**
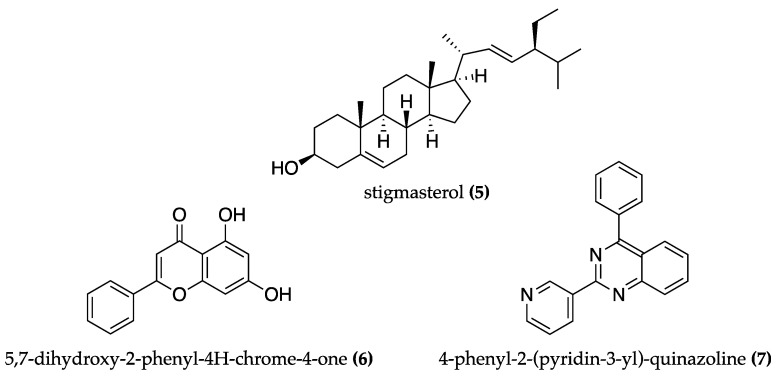
Structure of stigmasterol **(5)**, 5,7-dihydroxy-2-phenyl-4H-chrome-4-one **(6)**, and 4-phenyl-2-(pyridin-3-yl)-quinazoline **(7)**, which interact with AChE in silico.

A phytosterol-rich extract of *Phormidium autumnale* obtained through supercritical fluid extraction with ethanol (SFE-EtOH) revealed moderate to high inhibitory activity against AChE (IC_50_ = 65.80 μg/mL) and lipoxygenase (IC_50_ = 58.20 μg/mL) while showing a high antioxidant capacity (IC_50_ = 7.40 μg/mL). The presence of the phytosterol stigmasterol **(5)** (Figure 3) in the extract significantly correlates with AChE inhibition as it showed interactions with several AChE binding sites in molecular docking assays [60].

Refaay et al. (2022) [61] found that fraction 7 of the *Anabaena variabilis* methylene chloride/methanol (1:1) extract effectively reduced AChE activity (73.6%). This can be due to the presence of two aromatic compounds, the flavonoid 5,7-dihydroxy-2-phenyl-4H-chrome-4-one **(6)** and the alkaloid 4-phenyl-2-(pyridin-3-yl)-quinazoline **(7)**, shown in Figure 3, which interact with the allosteric binding site of AChE in molecular docking studies.

In another in vitro experiment, a crude methylene chloride/methanol (1:1) extract of *Oscillatoria sancta* lowered AChE activity by 60.7% [62]. The ethanolic extract of *Nostoc* sp. also showed significant inhibitory action against AChE (69.9%) at 3 mg/mL and against BChE (72.7%) at 5 mg/mL, as well as a high radical scavenging ability [63].

Other possible therapeutic targets include lowering the Aβ load, which can be accomplished by hindering Aβ formation [64]. Luo and Jing (2020) [65] showed that phycocyanin (0.5–50 μg/mL) from *Spirulina* sp. spontaneously inhibits the Aβ formation process of bovine serum albumin (BSA) by interacting in a gomphosis structure. Another study found that phycocyanin at a 5:1 (Aβ: phycocyanin) molar ratio had anti-amyloidogenic activity, as seen by its ability to inhibit Aβ40/42 fibrillation [66].

**Figure 4:**
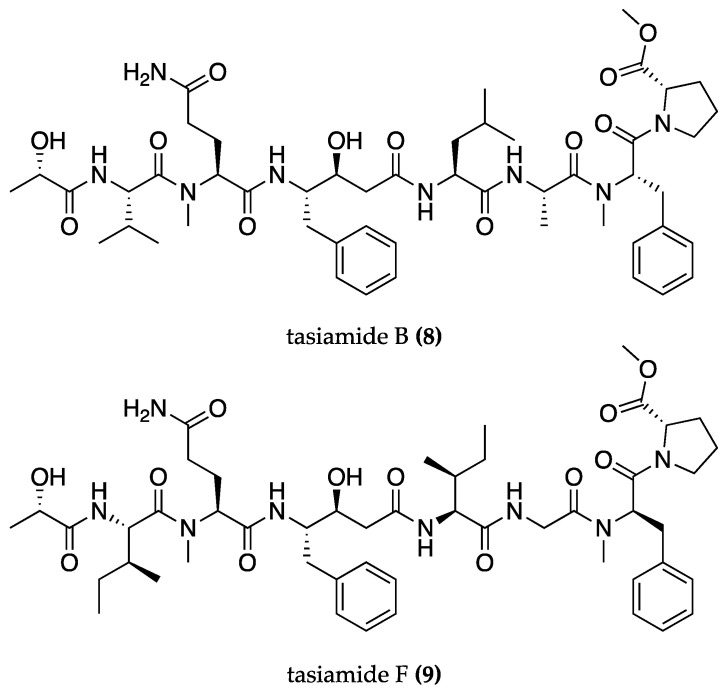
Structure of the cyanobacterial BACE-1 inhibitor tasiamide B **(8)** and its analog tasiamide F **(9)**.

The inhibition of the amyloidogenic pathway enzymes is an important strategy for reducing Aβ-peptide synthesis. This stops the conversion of APP into Aβ-peptide via sequential proteolytic cleavages by β-secretase 1 (BACE-1) and γ-secretase enzymes [64]. BACE-1 inhibitors derived from cyanobacteria have been identified, such as tasiamide B **(8)** (Figure 4) isolated from *Symploca* sp. [67,68] and its analog tasiamide F **(9)** (Figure 4) from *Lyngbya* sp. [69]. Tasiamide B (IC_50_ = 80 nM) is eight times more effective than tasiamide F (IC_50_ = 690 nM) due to modifications in the residues that engage in hydrophobic interactions with the receptor’s pocket and provide the inhibitory effect [69]. These can be the starting point for the design of more potent and selective BACE-1 inhibitors [67,68].

Phycobiliproteins from cyanobacteria also have potential as BACE-1 inhibitors. Molecular docking studies show that phycocyanin from *Leptolyngbya* sp. N62DM interacts with BACE-1 in an energetically favorable manner [70]. In the same study, an experiment was conducted using *Caenorhabditis elegans* CL4176, a transgenic model of AD that expresses Aβ_1–42_ in its muscle cells. It was found that phycocyanin administered through the medium (100 μg/mL) was able to rescue paralysis worms [70]. Similarly, Chaubey et al. (2019) [71] found that phycoerythrin from *Lyngbya* sp. A09DM exhibited significant interaction and binding affinity with BACE-1 in molecular docking studies and protein–protein interactions in vitro. These results were also further supported by in vivo experiments on *C. elegans* CL4176, where treatment with phycoerythrin (100 μg/mL) led to a reduction in Aβ deposition and senile plaque formation.

A study looked at the effects of oral pre-treatment with a 70% ethanol extract of *Spirulina maxima* (SM70EE) on rats with cognitive impairment caused by intracerebroventricular injection of Aβ_1–42_. The extract (150 and 450 mg/kg/day) decreased the levels of APP and BACE-1, thereby reducing APP processing and lowering Aβ accumulation in the hippocampus. It also improved cognition, reduced AChE activity, and suppressed hippocampal oxidative stress by improving the antioxidant system. The treatment stimulated the brain-derived neurotrophic factor (BDNF)/phosphatidylinositol-3 kinase (PI3K)/serine/threonine protein kinase (Akt) signaling pathway, which reduced glycogen synthase kinase-3 (GSK3β) phosphorylation, contributing to BACE-1 suppression [72].

**Figure 5:**
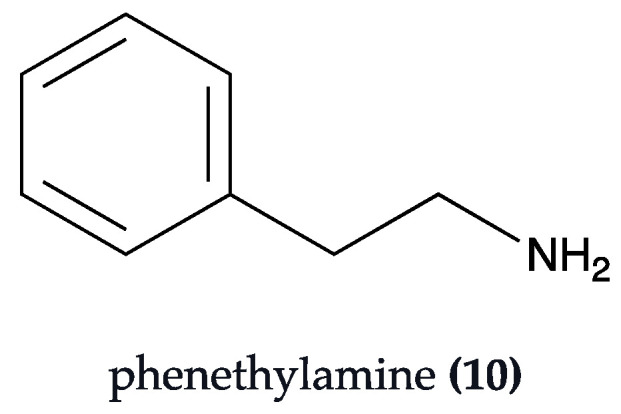
Structure of phenethylamine, one of the main components of the Klamin^®^ extract.

Galizzi et al. (2023) [73] studied the effects of KlamExtra^®^, a supplement derived from *Aphanizomenon flos-aquae*, in a high-fat diet rodent model of neurodegeneration. KlamExtra^®^ is a combination of the patented extracts Klamin^®^ and AphaMax^®^. Klamin^®^ contains a concentrated dose (15–18 mg) of phenylethylamine **(10)** (Figure 5), a compound that modulates both the nervous and immune systems, as well as phycocyanins, mycosporine-like amino-acids, and AFA-phytochrome, which are neuroprotectants and selective monoamine oxidase B inhibitors [74]. Additionally, AphaMax^®^ is rich in phycocyanins (25–30%) and polyphenols, which are powerful antioxidants, and anti-inflammatory molecules [75]. Specifically, polyphenols were also found to be involved in the regulation of autophagy in various NDs [76]. Treatment with KlamExtra^®^ (0.9 mg/mouse) induced a pattern of decreased BACE-1 and PSEN-1 expression, resulting in reduced APP processing and the accumulation of Aβ. It also safeguarded neural function and synaptic transmission by elevating synaptophysin levels and maintaining normal neuronal morphology. Furthermore, the extract improved the levels of metabolic markers related to glucose metabolism and showed anti-inflammatory properties by increasing IL-10 and modulating the astrocyte and microglia activation, with a decrease in the astrocyte marker glial fibrillary acid protein (GFAP) and an increase in soluble triggering receptor expressed on myeloid cells-2 (sTREM-2) [73]. Particularly, the increase in the immunosuppressive cytokine IL-10 has been described as promising in ND therapeutics, as recently reviewed [11].

Neurofibrillary tangles, which are composed of hyperphosphorylated tau protein, are also a hallmark of AD. Kinases, mainly GSK3β, are responsible for tau phosphorylation and thus, reducing enzymatic activity can reduce tau load [77]. In a study with Wistar rats treated with nicotine, a daily intraperitoneal injection with *S. platensis*-lipopolysaccharides (100 μg/kg) provided neuroprotection by suppressing the up-regulation of phosphorylated-tau ratio expression by two fold, while showcasing antioxidant, anti-inflammatory, and anti-apoptotic activities [78]. Dietary supplementation of 1% and 2% *Spirulina platensis* dry powder in high-fat diet mice lowered the tau burden by reducing both phosphorylated-tau and phosphorylated-GSK levels, while it also decreased Aβ_1–42_ concentrations, APP, and BACE-1 levels in the hippocampus [79].

**Figure 6:**
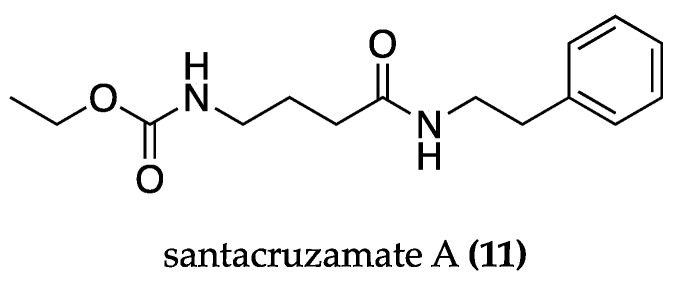
Structure of santacruzamate A **(11)**, a carbamate derivative with neuroprotective activity.

AD has also been linked to mitochondrial dysfunction and endoplasmic reticulum stress. Santacruzamate A **(11)** (Figure 6), a compound produced by a marine cyanobacterium cf. *Symploca* sp., has shown therapeutic potential in vitro and in vivo. It inhibited the Aβ_25–35_-induced apoptosis in PC12 cells (2 μM STA) by reversing the endoplasmic reticulum and unfolded protein response stress. It regulated the endoplasmic reticulum retention signal (KDEL) receptor, which increased chaperone luminal retention. Compound **11** also restored the mitochondrial intermembrane space assembly pathway and regulated the expression of the mitochondrial intermembrane space assembly protein 40 (Mia40) and the augmenter of the liver regeneration (ALR) system, resulting in a reduction in the mitochondrial fission and apoptosis pathways [80]. This was confirmed by in vivo studies in APPswe/PS1dE9 mice, a common AD mouse model bearing mutant transgenes of the amyloid precursor protein and presenilin-1, which lead to an early-onset increase in parenchymal Aβ-levels and other clinically relevant AD-like symptoms [81]. Treatment with santacruzamate A **(11)** (5 and 10 mg/kg/day) promoted memory performance in behavioral tests and enhanced KDELR and Mia40-ALR functions in the brain tissue [80].

Another pathological aspect of AD is heavy metal bioaccumulation and reversing its toxicity can improve disease outcomes. In Wistar rats, tablets of *S. platensis* (1500 mg/kg) revealed neuroprotective potential against brain degeneration induced by aluminum chloride (AlCl_3_). While lowering the number of illuminated Aβ protein aggregates, the treatment also reduced histopathological alterations in the cerebral cortex and hippocampus, with close to normal neuron morphology and fewer neurodegenerative features. It also improved metabolic indices and demonstrated anti-inflammatory activity through the reduction in TNF-α. The tablets showed strong antioxidant potential by decreasing thiobarbituric acid reactive substances (TBARS) levels and restoring glutathione (GSH) levels, thiol content, and total antioxidant capacity (TAC) [82].

In a study by Abdelghany et al. (2023) [83], an *S. platensis*-loaded niosome (SPLN) formulation was explored as a drug delivery system in an AlCl_3_-induced AD rat model. The use of nanoparticles enables more effective, controlled, and targeted brain treatment. *S. platensis*-loaded niosome (300 mg/kg) improved recognition and working memory and demonstrated neuroprotective activity by maintaining normal morphology in hippocampal brain tissue. Additionally, it restored AChE activity, ACh, and monoamine levels in the brain and also improved the oxidative state as it lowered the malondialdehyde (MDA) levels and TAC [83].

Growing data suggest that AD is associated with dysbiosis of the human gut microbiota via neuroinflammatory processes across the microbiota–gut–brain axis, suggesting that modifying the gut microbiota could be a strategy for treating the condition [84]. According to Zhou et al. (2021) [79], dietary supplementation with 1% and 2% *S. platensis* dry powder in high-fat diet mice alleviated cognitive impairment and restored gut microbial dysbiosis by increasing the Shannon, ACE, and Chao indices while decreasing the Simpson index, indicating enhanced microbial community richness and diversity. It improved the intestinal environment by balancing microbiota and increasing the abundance of beneficial microorganisms, such as *Verrucomicrobia*, while reducing the presence of harmful microorganisms, like *Firmicutes*. Supplementation also lowered inflammatory lipopolysaccharide levels in the feces and serum and raised fecal levels of short-chain fatty acids, which improves neuronal homeostasis. Furthermore, it showed anti-inflammatory benefits by lowering inflammatory markers such as GFAP, TNF-α, IL-1β, IL-6, and ionized calcium-binding adapter molecule 1 (IBA-1) in the hippocampus [79].

Aside from the modes of action outlined above, cyanobacteria, particularly *Spirulina* and its component phycocyanin, largely work through gene modulation.

In a study conducted in rodents intracerebroventricularly injected with Aβ_25–35_, the oral pre-treatment with a proteolysis product of phycocyanin (EDPC) from *S. platensis* (750 mg/kg) improved cognitive impairment in a Y maze spontaneous alternation test and modulated de gene expression profile in a DNA microarray analysis. It counteracted the aberrant expression of 35 genes, including Prnp, Cct4, Vegfd, Map9, Pik3cg, Zfand5, Endog, and Hbq1a, which are directly linked to AD or other neurological diseases [85].

In C57BL/6 mice injected with oligomeric Aβ_1–42_, treatment with phycocyanin (200 mg/kg) from *S. platensis* improved spatial memory and reversed the epigenetic dysregulation. It restored the expression of the regulatory miRNA-335, which was downregulated by 76%, and the expression of the BDNF gene, which was reduced to 24% in Aβ-mice. On the other hand, it downregulated the histone deacetylase 3 (HDAC3) gene, whose expression was amplified three fold in Aβ-mice. The treatment also showed anti-apoptotic and anti-inflammatory effects, by restoring Bax/Bcl-2 equilibrium, decreasing caspase-3 and caspase-9 release, and lowering inflammatory cytokine levels (IL-6 and IL-1β) [86].

Agrawal et al. (2020) [87] demonstrated that phycocyanin administration (100 mg/kg) in an intracerebroventricular streptozotocin-induced AD-mice model improved spatial memory and reduced memory impairment in behavioral tests. It improved metabolic parameters, by restoring the gene expression of insulin signaling molecules such as the insulin (INS) gene, insulin receptor substrate 1 (IRS-1), PI3K, and Akt. Thereby, it increased the activation of the insulin-PI3K-Akt pathway while it lowered the expression of one of its inhibitors, the phosphatase and tensin homolog (PTEN) gene. In addition, the treatment upregulated the anti-apoptotic marker Bcl-2 whereas the pro-apoptotic marker Bax was downregulated. It also altered acetylcholine metabolism by lowering AChE activity while increasing choline acetyltransferase (ChAT) in the hippocampus and mitigated neuroinflammation by reducing TNF-α and nuclear factor (NF)-kβ levels [87].

In another study, treatment with *S. platensis*-loaded niosome (300 mg/kg) modulated gene expression, restoring the mRNA levels of the enzymes AChE and monoamine oxidase and reversing both the AlCl_3_-induced decrease in the anti-apoptotic protein B-cell lymphoma-2 (Bcl-2) and increase in the pro-apoptotic protein Bcl-2 associated X-protein (Bax) mRNA levels [83].

**Table 1:** Cyanobacteria-derived products/extracts studied in AD disease models.

Strain	Compound/Extract	Effect	In Vitro Assays	In Vivo Assays	Reference
*Anabaena flos-aquae* NRC-525-17	Anatoxin-a(s) **(3)**	AChE and BChE inhibition	AChE and BChE inhibition assay		[55]
*Nostoc* 78-12A	Nostocarboline **(4)**	BChE inhibition	AChE and BChE inhibition assay		[57]
*Phormidium autumnale*	SFE-EtOH extract	AChE and LOX inhibition. Antioxidant.	AChE inhibition assay. LOX inhibition assay. ORAC assay.		[60]
*Anabaena variabilis*	Methylene chloride/ methanol extract (Fraction 7)	AChE inhibition	AChE inhibition assay		[61]
*Oscillatoria sancta*	Methylene chloride/ methanol (1:1) extract	AChE inhibition	AChE inhibition assay		[62]
*Nostoc* sp.	Ethanolic Extract	AChE and BChE inhibition. Antioxidant.	AChE and BChE inhibition assay. DPPH assay.		[63]
*Spirulina* sp.	Phycocyanin	Inhibition of Aβ formation	Fluorimetric assay. Kinetic analysis. Circular dichroism analysis.		[65]
*Spirulina* sp.	Phycocyanin	Inhibition of Aβ40/42 amyloid fibrillation	Fibrillar and amorphous aggregation assays. Transmission electron microscopy imaging.		[66]
*Symploca* sp.	Tasiamide B **(8)**	BACE-1 inhibition	BACE-1 inhibition assay		[67]
*Lyngbya* sp.	Tasiamide F **(9)**	BACE-1 inhibition	BACE-1 inhibition assay		[69]
*Leptolyngbya* sp. N62DM	Phycocyanin	BACE-1 inhibition	Protein-complex interface identification	*Caenorhabditis elegans* CL4176 transgenic AD-model: Paralysis assay	[70]
*Lyngbya* sp. A09DM	Phycoerythrin	BACE-1 inhibition	Surface plasmon resonance. Isothermal titration calorimetry. Enzyme activity by kinetic parameters.	*Caenorhabditis elegans* CL4176 transgenic AD-model: Thioflavin-T staining assay	[71]
*Spirulina platensis*	Lipopolysaccharide	Downregulation of p-tau expression. Antioxidant. Anti-inflammatory.		Wistar albino rats exposed to nicotine: Biochemical assessments (Oxidative and inflammatory markers). RT-PCR. Western Blot (p-tau).	[78]
*Spirulina maxima*	70% ethanol extract	AChE inhibition. Reduced Aβ, APP, and BACE-1 levels. BDNF/PI3K/Akt pathway activation. Antioxidant. Improved cognition.		ICR mice injected with Aβ_1–42_: Passive Avoidance Test. Morris WaterMaze Test. Biochemical Analysis (Aβ_1–42_, GSH, BDNF, AChE). Western Blot.	[72]
*Aphanizomenon flos-aquae*	KlamExtra^®^	Reduced Aβ, APP and BACE-1 levels. Anti-inflammatory and anti-gliosis. Improved metabolic parameters. Protection of neuronal morphology and synapses.		High-Fat Diet C57BL/6J mice: Metabolic parameters analysis. Western Blot (IR, Akt, PSEN-1, BACE-1, PSD-95, synaptophysin, TNF-α, GFAP, IL-10, TREM-2). Histopathology and Immunohistochemistry (GFAP, TREM-2, Aβ). Thioflavin T staining. TUNEL assay.	[73]
*Spirulina platensis*	Diet supplementation	Decreased Aβ_1–42_, APP, BACE-1, p-tau, and p-GSK levels. Anti-inflammatory. Improved microbiota dysbiosis. Improved metabolic parameters. Improved locomotor and cognitive function.		High-Fat Diet C57BL/6J mice: Barnes Maze test. Morris Water Maze test. ELISA (Aβ_1–42_, TNF-α, IL-1β, IL-6, LPS). RT-PCR. Western Blot (APP, BACE-1, p-tau, p-GSK, IBA-1). Microbial diversity analysis. GC (SCFAs).	[79]
cf. *Symploca* sp.	Santacruzamate A **(11)**	Anti-apoptotic. Anti-UPR and ER stress. Improvement of the mitochondrial fission pathway. Modulation of KDELR and Mia40-ALR. Memory improvement.	PC12 cells: Cell viability and apoptosis assays. Electrophysiological recordings. Immunoblot analyses. Measurement of mitochondrial permeability transition pore. Opening and mitochondrial membrane potentials.	APPswe/PS1dE9 mice: Open-Field test. Morris Water Maze test. RT-PCR (Mia40, KDEL).	[80]
*Spirulina platensis*	Diet supplementation (tablets)	Protection of neuronal morphology. Reduction in Aβ accumulation. Improvement of metabolic parameters. Antioxidant. Anti-inflammatory.		Wistar rats treated with AlCl_3_: TBARS assay. GSH content assay. Total thiol content assay. TAC assay. GPx, GST, SOD activity assay. Lipid profile determination. ELISA (TNF-α). Histology. Immunofluorescence (Aβ).	[82]
*Spirulina platensis*	*S. platensis*- loaded niosome	Protection of neuronal morphology. Restored levels of AChE and ACh. Gene modulation. Recognition and working memory improvement.		Wistar rats treated with AlCl_3_: Novel object recognition test. Y-maze test. TAC assay. MDA assay. AChE assay. Histology. HPLC (ACh, NE, 5HT, DA, DOPAC). qPCR (Bax, Bcl-2, AChE, MAO).	[83]
*Spirulina platensis*	Enzyme Digested Phycocyanin (EDPC)	Cognitive function improvement. Gene modulation.		Male Slc:ddY SPF mice injected with Aβ_25–35_: Y Maze test. DNA microarray.	[85]
*Spirulina platensis*	Phycocyanin	Gene and miRNA modulation. Anti-inflammatory. Anti-apoptotic. Memory improvement.		Male C57BL/6 mice injected with oligomeric Aβ_1–42_: Eight-arm radial maze. RT-PCR (caspase-3, caspase-9, miR-335). Western Blot (HDAC3, Bcl-2, Bax, IL-6, IL-1β). Immunohistochemistry (Bcl-2, Bax). Immunofluorescence (BDNF, HDAC3).	[86]
*Spirulina platensis*	Phycocyanin	AChE inhibition. ChAT activity increase. Gene modulation. Increased PI3K/Akt pathway. Anti-inflammatory. Memory improvement.		Female Wistar Rats injected with STZ: Morris Water Maze. Memory consolidation test. Novel object recognition test. Open field test. AChE and ChAT activity assays. ELISA (TNF-α, NF-kB p56, Bcl-2, Bax, BDNF, IGF-1). qRT-PCR (IRS-1, INS, PI3K, Akt, PTEN).	[87]
*Spirulina maxima*	70% ethanolic extract (SM70EE) pills	Memory and vocabulary improvement.		Randomized, double-blind, and placebo- controlled clinical trial. Visual learning, visual working memory, and verbal learning tests.	[88]
*Spirulina platensis*	Dietary supplementation	Improved cognitive function. Improved metabolic status.		Randomized, double-blind, and placebo. -controlled clinical trial. Mini-mental state exam. ELISA (hs-CRP, Insulin). Biochemical analysis (NO, TAC, GSH, MDA, FPG, lipid profile).	[89]

Abbreviations: AChE—Acetylcholinesterase. BChE—Butyrylcholinesterase. SFE-EtOH—Supercritical Fluid Extraction with Ethanol. LOX—Lipoxygenase. ORAC—Oxygen Radical Absorbance Capacity. DPPH—2,2-diphenyl-1-picrylhydrazyl. BACE-1—Beta Secretase 1. AD—Alzheimer’s Disease. Aβ—Beta-amyloid peptide. APP—Amyloid-beta Precursor Protein. BDNF—Brain-derived Neurotrophic Factor. PI3K—Phosphoinositide 3-kinase. Akt—Protein kinase B. TBARS—Thiobarbituric Acid Reactive Substances. GSH—Total Glutathione. IR—Insulin receptor. PSEN-1—Presenilin-1. PSD-95—Postsynaptic density protein 95. TNF-α—Tumor Necrosis Factor α. GFAP—Glial fibrillary acidic protein. IL-10—Interleukin. TREM-2—Triggering receptors expressed on myeloid cells-2. p-tau—Phosphorylated Tau. p-GSK—Phosphorylated Glycogen Synthase. LPS—Lipopolysaccharide. RT-PCR—Reverse Transcription Polymerase Chain Reaction. Iba-1—Ionized calcium-binding adaptor molecule 1. GC—Gas Chromatography. SCFAs—Short-chain fatty acids. UPR—Unfolded Protein Response. ER—Endoplasmic Reticulum. KDELR—Endoplasmic Reticulum Protein Retention Receptor. Mia40—Mitochondrial Intermembrane Space Assembly Protein 40. ALR—Augmenter of the Liver Regeneration. AlCl_3_—Aluminum Chloride. TAC—Total Antioxidant Capacity. GPx—Glutathione Peroxidase. GST—Glutathione S-transferase. SOD—Superoxide Dismutase. ELISA—Enzyme-Linked Immunosorbent Assay. MDA—Malondialdehyde. HPLC—High-Performance Liquid Chromatography. ACh—Acetylcholine. NE—Norepinephrine. 5HT—Serotonin. DA—Dopamine. DOPAC—3,4-Dihydroxyphenylacetic acid. Bcl-2—B-cell Lymphoma-2. Bax—Bcl-2 Associated X-protein. MAO—Monoamine oxidase. HDAC3—Histone deacetylase 3. STZ—Streptozotocin. ChAT—Choline acetyltransferase. NF-kβ—Nuclear Factor Kappa β. IGF-1- Insulin-like growth factor 1. IRS-1—Insulin receptor substrate 1. INS—Insulin Gene. PTEN—Phosphatase and Tensin Homolog. hs-CRP—High sensitivity C-reactive protein. NO—Nitric Oxide. FPG—Fasting Plasma Glucose. Human clinical trials have also validated the potential of *Spirulina* as a nutraceutical. Patients with mild cognitive impairment who consumed 1 g/day of *S. maxima* 70% ethanol extract (SM70EE) capsules experienced statistically significant gains in visual learning and working memory according to a randomized, double-blind, and placebo-controlled clinical trial [88]. Another randomized, double-blind, and controlled clinical trial investigated the cognitive and metabolic status of patients with AD who took *S. platensis* capsules twice daily (500 mg/day). Supplementation considerably improved the Mini-Mental State Examination score, indicating an enhancement in cognitive function. It also had a favorable impact on the metabolic status by lowering C-reactive protein, fasting glucose, insulin levels, and insulin resistance while increasing insulin sensitivity [89].

### 4.2. Cyanobacteria against Parkinson’s Disease

Parkinson’s Disease (PD) is a neurodegenerative movement disorder whose incidence and prevalence increase with age. It is distinguished by the presence of Lewy bodies, which are intracellular protein aggregates of misfolded *α*-synuclein (*α*-Syn) protein, as well as the gradual loss of dopaminergic nigrostriatal neurons in the midbrain substantia nigra pars compacta [90]. Other features of PD include decreased dopamine metabolism impaired mitochondrial function, autophagy failure, oxidative stress, inflammation, and accelerated apoptosis. These lead to symptoms like decreased motor function, bradykinesia, postural instability, and muscle rigidity [91,92]. PD can be caused by many factors, including environmental exposure and genetics/epigenetics, with the most common mutations being in SNCA, LRRK2, PRKN, PINK1, and GBA genes [90].

There is no cure for PD but some treatments do exist to manage its symptoms. Most options aim to increase dopamine levels, including dopaminergic drugs such as levodopa, and enzyme inhibitors of monoamine oxidase B (MAO-B) and catechol-O-methyltransferase (COMT). Antioxidants, anti-inflammatory drugs, gene therapies, stem cell treatments, and protein aggregation inhibitors can be future therapeutic approaches [7].

Regarding cyanobacteria, the most compelling evidence for its use against PD comes from research on *Spirulina* and phycobiliproteins, namely phycocyanin (Table 2). This molecule can reduce the synucleinopathy typical of PD. At a molar ratio of 2:1 (α-Syn: phycocyanin), it was found to be an efficient inhibitor of A53Tα-synuclein amyloid fibrillation in silico. Interactions between phycocyanin and α-Syn were unstable, implying that brief interactions may limit fibril formation [66].

Macedo et al. (2017) [93] studied the effects of phycocyanin from *S. platensis* in a yeast model of PD transformed with a plasmid carrying the human gene of α-Syn. The phycocyanin-supplemented medium (48 mg/mL) promoted cell viability while drastically decreasing the fraction of cells exhibiting αSyn-GFP inclusions. It significantly reduced oxidative stress by lowering superoxide levels and lipid peroxidation, while enhancing thiol levels and catalase activity. Phycocyanin also displayed gene modulation properties. It ameliorated the oxidative stress response by modulating SOD1, SOD2, HAP4, and GLR1 genes and improved proteostasis by restoring RPN4 and ATG8 transcript levels while decreasing HSP26 mRNA levels, all of which are key players in proteosomal and autophagic activity.

Another distinguishing aspect of PD is low dopamine (DA) levels. To ameliorate the disease severity and reduce typical motor symptoms, it is vital to find therapeutic solutions that safeguard the levels of DA and its metabolites in the synaptic cleft [94]. Oral pre-treatment with *S. maxima* (150 mg/kg) partially protected dopamine depletion by 51%, and blocked lipid peroxidation by 100% in C-57 mice subjected to 1-methyl-4-phenyl-1,2,3,6-tetrahydropyridine (MPTP) neurotoxicity, which is a common chemically induced PD animal model [95].

According to Tóbon-Velasco et al. (2013) [96], rats injected with 6-hydroxydopamine (6-OHDA), another common animal model of chemically induced PD, and fed a diet supplemented with *S. maxima* (700 mg/kg/day) showed partial protection in the levels of DA (31%), homovanillic acid (47%), and 3,4-dihydroxyphenylacetic acid (23%) in the striatum. The treatment improved locomotor function, including greater use of both forelimbs and decreased circling behavior. It also enhanced antioxidation by reducing ROS production by 112% and nitrite levels by 77%, as well as considerably lowering lipid peroxidation and mitochondrial reduction activity [96].

In another study using a 6-OHDA-induced PD rat model, treatment with an aqueous freeze-dried extract of *Spirulina fusiform* (500 mg/kg, twice daily) resulted in a positive response in behavioral and motor tests, DA levels, and oxidative state. Moreover, the treatment in conjunction with amantadine, a medication commonly used to treat dyskinesia in PD patients, resulted in a significant increase in DA levels, a recovery of glutathione levels, and a reduction in TBARS content by 73% [97].

According to Xu et al. (2023) [98], three novel peptides (MAAAHR, MPQPPAK, and MTAAAR), derived from phycocyanin from *S. platensis*, showed significant neuroprotective activity in MPTP-induced PD zebrafish. The peptides (12.5 μg/mL, 25 μg/mL, and 50 μg/mL) relieved locomotion constraints and reversed the DA neuron degeneration and neural vasculature disorganization. Furthermore, they increased antioxidant enzyme activity (SOD, CAT, and GSH-Px) while decreasing ROS and protein carbonyl levels. They also had anti-apoptotic effects, lowering the number of apoptotic brain cells and the activity of AChE, which is involved in apoptotic pathways. The observed effects can be attributed to the modulation of gene expression as they upregulated oxidative stress response genes (nrf2, ho-1, nqo-1, gclc, and gclm) and downregulated genes linked to autophagy (α-syn, parkin, beclin1, atg5, map1lc3b, and atg3) and apoptosis (caspase-1, caspase-3, caspase-8, caspase-9, and Bax) [98].

*Drosophila melanogaster* flies are a common animal model of PD [99]. Treatment with *S. platensis* methanolic extract (120 μg/mL) in *D. melanogaster* subjected to FeSO_4_ toxicity, improved the survival rate and locomotor ability of the flies. It promoted an increase in DA levels and showed strong antioxidant activities by scavenging DPPH free radicals (IC_50_ = 64.55 μg/mL) and reducing MDA levels [100]. Another study explored the effects of *Spirulina* supplementation (5% or 10%) in DJ-1β^Δ93^ flies exposed to chemically induced oxidative stress using paraquat. This is a transgenic model of PD, in which the loss of the *DJ-1*β ortholog gene improves vulnerability to oxidative stress and leads to dopaminergic neuronal degeneration [99]. The mixed diet with *Spirulina* significantly increased the locomotor capacity and the lifespan of the flies and improved the antioxidant state by downregulating the SOD/CAT activity. The authors also studied the effects of a phycocyanin-mixed diet (1 or 2 μg/mL). Both *Spirulina* and phycocyanin reduced cellular stress, as evidenced by a decrease in the expression of heat shock protein 70 (HSP70) and Jun-N-terminal kinase (JNK) expression [101].

Some of the studies present in the literature refer to the effect of cyanobacteria on the tyrosine hydroxylase (TH) levels, since this is the limiting enzyme in dopamine synthesis, and on the DA transporter (DAT), which regulates DA reuptake. Both TH and DAT expression is reduced in PD [102].

A study directed to evaluate the pre-treatment with intraperitoneal injections of a polysaccharide derived from *S. platensis* (800 mg/kg) in MPTP-treated mice revealed a significant increase in the DAT binding ratio and the TH-immunoreactive neurons in the *substantia nigra pars compacta*, along with their mRNA expression. It also showed strong antioxidant capacity, with increased serum levels of superoxide dismutase (SOD) and glutathione peroxidase (GSH-Px) [103]. Also, in rats subjected to an intrastriatal injection of 6-OHDA, treatment with a protein-enriched fraction of *S. platensis* (10 mg/kg) improved behavioral assessments. It also promoted the reversal of the 6-OHDA-induced decrease in striatal dopamine and 3,4-dihydroxyphenylacetic acid levels, while it significantly protected the striatal expression of TH and DAT. By lowering brain nitrite levels and lipid peroxidation, as well as the expression of GFAP, hippocampus inducible nitric oxide synthase (iNOS), and cyclooxygenase-2 (COX-2) enzymes, the fraction demonstrated antioxidant and anti-inflammatory potential [104]. Similarly, Lima et al. (2017) [105] showed that treatment with 10% (*w*/*v*) aqueous extract of *S. platensis* at 25 mg/kg or 50 mg/kg in rats subjected to 6-OHDA-induced parkinsonism is neuroprotective. Both treatments improved apomorphine-induced rotational behavior, reversed the reduction in dopamine and 3,4-dihydroxyphenylacetic acid levels in the striatum, and showed antioxidant activity by reducing nitrite levels and inhibiting lipid peroxidation. Treatment at 50 mg/kg partially blocked the decrease in TH (42%) and DAT immunoreactivity (55%) and exhibited anti-inflammatory activities, as seen by the decrease in iNOS and COX-2 immunoreactivity, two enzymes related to inflammation [105].

Pre-treatment with a 0.1% *Spirulina*-supplemented diet in a rat model of PD inoculated with an adeno-associated virus for α-Syn protected against neuronal loss, as seen by the increase in TH-positive and NeuN-positive neurons in the substantia nigra pars compacta. It also showed anti-inflammatory activity, with a decrease in the number of activated microglial cells, as evaluated by a reduction in OX-6-(MHC class II)-positive immunoreactivity and a significant increase in the expression of the fractalkine receptor (CX3CR1) in microglia that, when stimulated, promotes anti-inflammatory activities [106]. On the same note, Strömberg et al. (2005) [107] showed that a diet enriched with 0.1% *Spirulina* fed to rats injected with 6-OHDA promoted the recovery of striatal dopamine innervation and positive TH nerve fibers, driven by an early and temporary increase in OX-6-(MHC class II) positive microglia that induce remodulation.

In PD, a strong association between DA neurodegeneration and inflammation has been described with the involvement of several inflammatory mediators and cells, such as peripheral immune cells. Hence, the increase in TH and DAT induced by *Spirulina*, as described before, might not be due to a direct effect on the production of the enzyme and DAT but instead due to anti-inflammatory and antioxidant responses. In fact, several results support that in CNS, DA depletion modulates peripheral immunity and expression of the dopaminergic markers TH and DAT on peripheral immune cells [99,100,101,102,103,104,105,106].

**Table 2:** Cyanobacteria-derived products/extracts studied in PD disease models.

Strain	Compound/Extract	Effect	In Vitro Assays	In Vivo Assays	Reference
*Spirulina* sp.	Phycocyanin	Inhibition of A53Tα-synuclein amyloid fibrillation	Fibrillar and amorphous aggregation assays. Transmission electron microscopy imaging.		[66]
*Spirulina platensis*	Phycocyanin	Reduction in α-synuclein inclusions. Gene modulation. Antioxidant. Improved proteostasis.		BY4741 Yeast transformed with p42FAL-αsyn-GFP: Spot assay. Fluorescence microscopy. Western Blot (α-syn). Flow cytometry. TBARS assay. CAT activity. Total thiols assay. qRT-PCR (SOD1, SOD2, HAP4, LHS1, HRD1, GSH1, GLR1, RPN4, ATG8).	[93]
*Spirulina platensis*	Phycocyanin derived peptides (MHLWAAK, MAQAAEYYR, MDYYFEER)	Improved locomotion. Neuronal protection. Antioxidant. Anti-apoptosis. Gene modulation.		MPTP-induced parkinsonism in transgenic zebrafish: Fluorescence Microscopy. Behavioral tests. Fluorescence ROS determination. Biochemical analysis (SOD, CAT, GSH-Px, CO, AChE). Acridine orange staining. qRT-PCR.	[98]
*Spirulina maxima*	Diet supplementation	Protection of DA and HVA content. Blockage of lipid peroxidation.		MPTP-induced parkinsonism in male C-57 rats: HPLC (DA, HVA, 5-HIAA, 5-HT). TBARS Assay.	[95]
*Spirulina maxima*	Diet supplementation	Improved locomotion. Recovery of mitochondrial activity. Protection of DA, DOPAC, and HVA levels. Antioxidant.		6-OHDA-induced parkinsonism in male Wistar rats: Turn-behavior test. Closed-field test. Cylinder test. Fluorescence ROS determination. Griess reaction. TBARS assay. MTT assay. HPLC (DA, DOPAC, HVA).	[96]
*Spirulina fusiform*	Aqueous freeze-dried extract suspended in olive oil	Improved behavior and locomotion. Protection of DA levels. Antioxidant.		6-OHDA-induced parkinsonism in male Wistar albino rats: Amphetamine- and Apomorphine -induced rotations. Locomotor activity. Rota rod. TBARS assay. Reduced glutathione content assay. HPLC (DA).	[97]
*Spirulina platensis*	Methanolic extract	Increased lifespan and locomotion. Antioxidant. Protection of DA content.		*Drosophila Melanogaster* exposed to FeSO_4_: Total phenol Content. DPPH radical scavenging activity. Survival rate. Negative Geotaxis assay. Lipid Peroxidation Assay. DA content assay.	[100]
*Spirulina platensis*	Diet supplementation	Increased lifespan and locomotion. Antioxidant. Reduced cellular stress.		DJ-1β^Δ93^ *Drosophila Melanogaster* exposed to paraquat: Survival assay. Locomotor assay. PCR (HSP70). SOD and CAT enzymatic assays. Immunostaining (Hsp70 and JNK).	[101]
*Spirulina platensis*	Polysaccharide	Increased TH and DAT expression. Antioxidant.		MPTP-induced parkinsonism in male C57BL/6J mice: Immunohistochemistry and RT-PCR (TH, DAT). SOD and GSH-Px assays.	[103]
*Spirulina platensis*	Protein-rich fraction (SPF)	Improved behavior. Protection of DA and DOPAC levels. Increased TH and DAT expression. Reduced iNOS, COX-2, and GFAP expression. Antioxidant.		6-OHDA-induced hemiparkinsonism in male Wistar rats: Apomorphine-induced rotational test. Open-field test. Forced swim test. HPLC (DA, DOPAC). Griess Reaction. TBARS assay. Immunohistochemistry (TH, DAT, iNOS, GFAP, COX-2)	[104]
*Spirulina platensis*	10% (*w*/*v*) aqueous extract	Improved behavior. Protection of DA and DOPAC levels. Protection of TH and DAT expression. Decreased iNOS and COX-2. Antioxidant.		6-OHDA-induced parkinsonism in male Wistar rats: Apomorphine-induced rotational test. HPLC (DA, DOPAC). Griess Reaction. TBARS assay. Immunohistochemistry (TH, DAT, iNOS, COX-2).	[105]
*Spirulina*	Diet supplementation	Increase in TH^+^ and NeuN^+^ neurons. Anti-inflammatory.		F344 rats treated with AAV9α-synuclein: Immunohistochemistry (TH, α-synuclein, OX-6, NeuN). Stereology. Western Blot (CX3CR1).	[106]
*Spirulina*	Diet supplementation	Recovery of striatal dopamine innervation. Increased TH^+^ fibers. Anti-inflammatory.		6-OHDA-induced parkinsonism in F344 male rats: Immunohistochemistry (TH, OX-6, Iba1, GFAP). Cell counting.	[107]

Abbreviations: α-syn—α-synuclein. GFP—Green fluorescent protein. TBARS—Thiobarbituric acid reactive substances. CAT—Catalase. RT-PCR -Reverse transcription polymerase chain reaction. SOD1—Copper-zinc superoxide dismutase. SOD2—Manganese superoxide dismutase. HAP4—Glucose-repressed regulated subunit of the HAP transcriptional complex. LHS1—Heat shock protein 70 homolog. HRD1—E3 ubiquitin-protein ligase. GSH1—Gamma-glutamylcysteine synthetase. GLR1—Glutathione reductase. RPN4—Zinc-coordinating proteasomal transcription factor. ATG8—Autophagy-related protein 8. DA—Dopamine. HVA—Homovanillic acid. MPTP—1-methyl-4-phenyl-1,2,3,6-tetrahydropyridine. HPLC—High-performance liquid chromatography. 5-HIAA—5-Hydroxyindoleacetic acid. 5-HT—5-hydroxytryptamine. DOPAC—3,4-Dihydroxyphenylacetic acid. 6-OHDA—6-hydroxydopamine. ROS—Reactive oxygen species. MTT—3-(4,5-dimethylthiazol-2-yl)-2,5-diphenyl-2H-tetrazolium bromide. TH—Tyrosine hydroxylase. DAT—Dopamine transporter. SOD—Superoxide dismutase. GSH-Px—Glutathione peroxidase. iNOS—Inducible nitric oxide synthase. COX-2—Cyclooxygenase 2. GFAP—Glial fibrillary acidic protein. NeuN—Neuronal nuclear protein. OX-6—Major histocompatibility complex (MHC) class II antigen. CX3CR1—Fractalkine receptor. Iba1—Ionized calcium-binding adaptor. FeSO4—Ferrous sulfate. DPPH—2,2-diphenyl-1-picrylhydrazyl. HSP70—70 kilodalton heat shock protein. JNK—c-Jun N-terminal cinase. CO—Protein carbonyl. AChE—Acetylcholinesterase.

### 4.3. Cyanobacteria against Multiple Sclerosis

Multiple Sclerosis (MS) is a chronic and inflammatory ND with an autoimmune origin that affects the CNS of more than two million people worldwide [108]. The main pathological hallmark is focal plaques, which are areas of immune cell infiltration and demyelination in the white and grey matter, that can be found in the brain, optic nerve, and spinal cord. These contribute to axon loss, myelin sheath destruction, and neuronal impairment. Other pathological processes include immune dysfunction, blood–brain barrier permeability, mitochondrial dysfunction, and oxidative injury. This can result in several symptoms, including visual loss, muscle weakness, balance problems, and cognitive impairment [109,110]. Although the specific causes of MS are unknown, genetic polymorphisms, particularly in the genes encoding human leukocyte antigen (HLA), lifestyle, and environmental factors are considered to play a role [110].

There is no cure for MS and most treatments revolve around immunomodulation, like interferon-beta. Research is being conducted to uncover new immune modulation targets and other strategies, such as remyelinating and cell-based therapies [108]. Preserving the normal functioning of the immune and inflammatory systems, decreasing oxidative stress, and maintaining neuronal integrity are crucial objectives in MS treatment. In this context, Spirulina-derived compounds such as phycocyanin and its tetrapyrrolic prostate group, phycocyanobilin, have been thoroughly studied [14] (Table 3).

Most research is conducted in experimental autoimmune encephalomyelitis (EAE) rodent models. EAE is an induced inflammatory disease of the CNS, where the immune system becomes activated in response to self-antigens, resulting in a pathology that is similar to that of MS [111]. Pentón-Rol et al. (2011) [112] investigated the prophylactic and therapeutic effects of phycocyanin (25 mg/kg/day) from S. platensis in an EAE model. The prophylactic schedule prevented disease development and both schedules ameliorated the mean cumulative score. The treatments provided neuronal protection as rats showed compressed, solid, and squashed myelin and no signs of axonal breakdown. It also attenuated protein and lipid damage, as evidenced by the reduced levels of MDA, advanced oxidation protein products (AOPP), peroxidation potential (PP), and ferric-reducing ability of plasma (FRAP) [112]. In peripheral blood mononuclear cells (PBMC) from MS patients, stimulation with phycocyanin induced a regulatory T cell (Treg) response, by increasing the expression of all Treg cell markers, including CD25, Foxp3, TGF-β, and IL-10, and the number of CD4^+^CD25^high^Foxp3^+^ T cells, indicating an ability to induce the Treg subset, which is reduced in MS patients [112].

**Figure 7:**
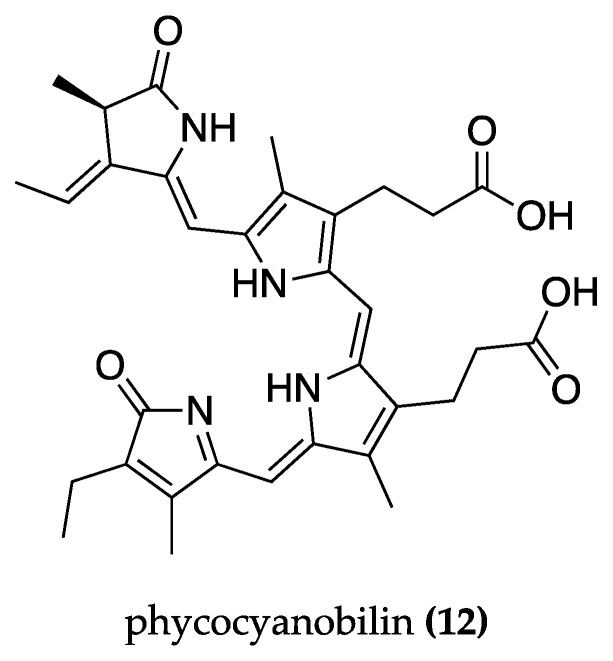
Structure of phycocyanobilin **(12)**, a linear tetrapyrrole chromophore covalently attached to protein subunits of phycocyanin.

In another study, the effects of phycocyanin from *S. platensis* and its tetrapyrrolic prostate group, phycocyanobilin **(12)** (Figure 7), were investigated. In EAE-Lewis rats, the prophylactic regimen of oral phycocyanin administration (200 mg/kg) eliminated disease symptoms, whereas the therapeutic regimen (200 mg/kg) significantly reduced the maximum clinical score and delayed disease onset. Both regimens produced a positive effect on motor impairment. Phycocyanin exhibited antioxidant activity by lowering MDA, PP, and FRA levels in serum and preserved myelin integrity, as evidenced by transmission electron microscopy, which revealed that rats treated with phycocyanin had compressed, solid, and squashed myelin. In the same study, oral phycocyanobilin (5 mg/kg) treatment in EAE-C57BL/6 mice improved clinical progression and reduced neuroinflammation by significantly lowering brain expression of IL-6 and IFN-γ, which are pro-inflammatory cytokines implicated in MS pathology [113].

A therapeutic regimen of phycocyanin (4 or 8 mg/kg) from *S. platensis* decreased disease severity and improved clinical performance in an EAE-mice model. It protected against demyelination and axonal degeneration, as evidenced by a lesser extent of demyelination in the spinal cord and a decrease in the density of APP-positive axons in white matter. It also demonstrated anti-inflammatory activity as it reduced inflammatory foci in the spinal cord, decreased Mac-3 activated microglia and CD3-positive T cells in lesions, and down-regulated Foxp3, a regulatory T cell marker, in the brain. The treatment also reduced the expression of IL-17 mRNA in the brain and serum, the main effector cytokine in MS, as well as other pro-inflammatory cytokines (IL-6). It was also efficient in lowering oxidative stress, as measured by lower levels of MDA, PP, and the CAT/SOD ratio [114].

Phycocyanin’s therapeutic effects can be linked to its gene-modulatory properties. In the same study by Pentón-Rol et al. (2016) [114], phycocyanin (8 mg/kg) modulated the expression of 918 genes, with prominence in the upregulation of genes involved in remyelination, gliogenesis, and axon–glia interactions (Mal, Mog, Mobp, Nkx6-2, Nkx2-2, Bmpa, and the transcription factor Olig1) while decreasing the expression of genes implicated in demyelination, such as CD44 and PPARMal. The authors also compared the effects of phycocyanin treatment to those of the standard treatment for MS, interferon-beta (IFN-β). While both had antioxidant and anti-inflammatory effects and modulated some of the same genes, they differed in several specific biological processes, implying that the combined treatment may provide additional benefits [114].

Gardón et al. (2022) [115] investigated the effects of phycocyanobilin **(12)** on gene modulation in vitro in a SH-SY5Y cell line model of glutamate-induced excitotoxicity. Pre-stimulation with phycocyanobilin (0.1 M) promoted a significant downregulation of the CYBB, HMOX-1, and HIF1A genes, all of which are linked to neurodegenerative diseases. On the other hand, it led to an increase in the expression levels of the genes encoding the detoxifying enzymes SOD2 and CAT, the antioxidant gene GPX1, the apoptosis-related proteins Bax and Bcl-2, and the transcription factor NFBK1. They also investigated the effects of oral phycocyanobilin treatment (1 mg/kg) in an EAE-induced MS rat model, which showed a tendency to reduce clinical signs as well as significantly lower the levels of pro-inflammatory cytokines (IL-17, IFN-γ, and IL-6) [115].

Similarly, in a MOG_35–55_ induced EAE rat model, treatment with phycocyanobilin **(12)** (0.5 and 1 mg/kg) showed protection against MS, delaying the disease’s symptoms and severity. The treatment regulated gene expression by down-regulating the TNF-α, LINGO1, and NOTCH1 genes, which mediate myelin damage, and by up-regulating the CXCL12, MOG, NKX2-2, OLIG1, and MAL genes, which mediate myelin protection. Phycocyanobilin reduced demyelination in the white matter of the spinal cord and showed anti-inflammatory properties as it decreased the levels of the pro-inflammatory cytokines IL-17A and IL-6 and the number of microglia/macrophages, marked by the diminution of Mac-3 and CD3 expression. It also increased the number of oligodendrocyte precursor cells and mature oligodendrocytes as shown by a significant increase in both oligodendrocyte markers (Olig2 and TPPP/p25) while decreasing APP levels, a marker of axonal damage [116].

**Table 3:** Cyanobacteria-derived products studied in MS disease models.

Strain	Compound/Extract	Effect	In Vitro Assays	In Vivo Assays	Reference
*Spirulina platensis*	Phycocyanin	Decreased the mean cumulative score. Neuronal Morphology Protection. Antioxidant. Anti-inflammatory. Treg induction.	PBMCs: RT-PCR (TGF-β, IL-10, CD25, Foxp3). Flow cytometry (CD4, CD25, CD69).	EAE induction in male Lewis rats: MDA assay. PP assay. TOP assay. AOPP assay. FRAP assay. Transmission electron microscopy studies.	[112]
*Spirulina platensis*	Phycocyanin	Improvement in disease onset and locomotion. Neuronal Morphology Protection. Antioxidant. Anti-inflammatory.		EAE induction in male Lewis rats and female C57BL/6 mice: Rotarod test. MDA assay. PP assay. FRA assay. Transmission electron microscopy studies. ELISA (IL-17, IL-6, IFN-γ).	[113]
*Spirulina platensis*	Phycocyanin	Improvement in disease onset. Antioxidant. Anti-inflammatory. Anti-demyelination. Neuronal Protection. Gene Modulation.		EAE induction in C57BL/6 mice: Immunohistochemistry (CD3, Mac-3, APP). Morphometric Analysis. MDA assay. PP assay. SOD, CAT, and GSH assays. IL-17 quantification. RT-PCR. Microarray Analysis.	[114]
*Spirulina platensis*	Phycocyanobilin **(12)**	Improvement in disease onset. Anti-inflammatory. Antioxidant. Anti-apoptosis. Gene modulation.	Human SHSY5Y cells: RT-PCR. Gene expression profile analysis.	EAE induction in C57BL/6 mice: ELISA (IL-17, IL-6, IFN-γ). Transmission electron microscopy. Immunohistochemistry (caspase-3, CD11).	[115]
*Spirulina*	Phycocyanobilin **(12)**	Improvement in disease onset. Anti-inflammatory. Anti-demyelination. Neuronal protection. Gene modulation.	T_MBP-GFP_ cells: Proliferation assay. Fluoresce microscopy.	EAE induction in C57BL/6 mice: Immunohistochemistry (CD3, Mac-3, APP, TPPP/p25, Olig2). Morphometric analysis. ELISA (IL-17A, IL-6, and IL-10). qPCR. Flow cytometry.	[116]

Abbreviations: PBMCs—Human peripheral blood mononuclear cells. RT-PCR—Reverse transcription polymerase chain reaction. TGF-β—Transforming growth factor beta. CD25—Cluster of differentiation 25. FOXP3—Forkhead box P3. CD4—Cluster of differentiation 4. CD69—Cluster of Differentiation 69. EAE—Experimental autoimmune encephalomyelitis. MDA—Malondialdehyde. PP—Lipid peroxidation. TOP—Total organoperoxides. AOPP—Advanced Oxidation of Protein Products. FRAP—Ferric Reducing Ability of Plasma. FRA—Ferric Reducing Ability. IL—Interleukin. IFN-γ—Interferon gamma. ELISA—Enzyme-Linked Immunosorbent Assay. CD3—Cluster of Differentiation 3. APP—Amyloid-beta precursor protein. SOD—Superoxide dismutase. CAT—Catalase. GSH—Total Glutathione. CD11—Cluster of Differentiation 11. TPPP/p25—Tubulin polymerization-promoting protein. Olig2—Oligodendrocyte transcription factor.

### 4.4. Cyanobacteria against Amyotrophic Lateral Sclerosis

Amyotrophic lateral sclerosis (ALS), also known as Lou Gehrig’s disease, is a rare and fatal ND that affects motor neurons, with an estimated global incidence of 2 per 100,000 person each year [117]. It is characterized by the degeneration of upper motor neurons in the brain’s motor cortex and the loss of lower motor neurons in the brainstem and spinal cord. The most significant neuropathological findings are intracellular cytoplasmic aggregates of eosinophilic Bunina bodies and ubiquitinated TDP-43 protein. Excitotoxicity, mitochondrial dysfunction, oxidative stress, inflammation, decreased axonal transport, and faulty RNA and DNA metabolism are all implicated in ALS pathophysiology. It causes muscle weakening, spasticity, and atrophy, which results in movement, speech, and breathing difficulties, finally leading to paralysis and death from respiratory failure within two and three years [118,119].

Although the cause of ALS is unknown, genetic mutations, especially in the SOD1, C9orf72, TARDB, and FUS genes, or environmental factors, can be involved [118,119].

There is currently no cure for ALS and the available treatments merely provide symptomatic alleviation. Medications like riluzole and edaravone can delay disease progression and improve survival but they cannot reverse the damage. Gene therapy, stem cells, or antibodies may be explored as future treatments [120].

However, to a much lesser extent, cyanobacteria-derived compounds have also been shown to be beneficial in the treatment of ALS (Table 4).

De Paola et al. (2012) [121] investigated the in vitro and in vivo effects of VB3323, a highly (95%) purified form of cyanobacterial LPS-like molecule (CyP) isolated from *Oscillatoria planktothrix* sp. Co-treatment with VB3323 (20 μg/mL) after LPS inhibited cell activation and morphological changes to the reactive phenotype in purified cultures of microglia. This molecule also significantly reduced the release of pro-inflammatory cytokines (TNF-α, IL-1β, and IL-6) induced by LPS in co-cultures of motor neuron cells and microglia and restored motor neuron viability (91.3%), counteracting LPS and its bioactive form lipid A toxicity. Using an in vivo model of motor neuron degeneration (Wobbler mice), the treatment with VB3323 intraperitoneally injected three times a week (0.5 mg/mL) slowed the disease progression and improved motor behavioral scores. It also reversed the morphological changes in motor neurons while reducing GFAP immunoreactivity and TNF-α expression in the ventral horn of the cervical spinal cord [121].

In an in silico experiment, β-carotene, chlorophyll-a, chlorophyll-b, phycoerythrin, and phycocyanin, which are the main natural pigments in cyanobacteria, were docked against the p75 neurotrophin receptor, the EphA4 receptor, and the HDAC receptor, which are promising therapeutic targets in ALS. It was discovered that β-carotene, phycoerythrin, and phycocyanin had high binding energies to the targets, indicating possible antagonistic activity [122].

A study explored the effects of a diet supplemented with 0.1% *Spirulina* for 10 weeks in a SOD1^G93A^ mice model of ALS. This model overexpresses the human SOD1 protein containing the G93A mutation, which is common in human familial ALS, and exhibits similar clinical and neuropathological findings of ALS [123]. The supplementation with *Spirulina* promoted extension reflex maintenance, particularly in the right hindlimbs, delaying the development of symptoms. It reduced motor neuron degeneration in the lumbar spinal cord, with fewer FluorJade-labeled neurons, a marker of degeneration, and fewer activated astrocytes marked by GFAP. It also had anti-inflammatory properties, reducing the levels of pro-inflammatory cytokines like IL-1β and TNF-α in the brainstem [124].

**Table 4:** Cyanobacteria-derived products/extracts studied in ALS disease models.

Strain	Compound/Extract	Effect	In Vitro Assays	In Vivo Assays	Reference
*Oscillatoria planktothrix* sp.	LPS-like molecule VB3323	TLR4 antagonist. Improved motor function tests. Anti-inflammatory and anti-gliosis. Neuroprotection.	Purified microglial cells: Immunocytochemistry (CD11b). Immunoblotting (CD68). Live cell imaging (GFP) Motor neurons/glia co-culture: ELISA (TNF-α, IL-1β and IL-6). Motor neurons/glia coculture and purified motor neurons: Motor Neuron Viability Assay (SMI32).	Wobbler Mice: Paw abnormality and grip strength test. Immunohistochemistry (GFAP, CD11, and TNF-α).	[121]
*Spirulina*	Diet supplementation	Maintenance of extension reflex. Anti-inflammatory. Neuroprotection against motor neuron degeneration.		SOD1^G93A^ mice: Weight and measurement. Extension Reflex test. Ribonuclease Protection Assay (IL-1α, IL-1β, IL-6, TNF-α). Immunohistochemistry (Fluoro-Jade, GFAP)	[124]

Abbreviations: IL—Interleukin. TNF-α—Tumor necrosis factor-alpha. GFAP—Glial fibrillary acidic protein. TLR4—Toll-like receptor 4. CD11—Cluster of Differentiation 11. CD68—Cluster of Differentiation 68. GFP—Green fluorescent protein. ELISA—Enzyme-Linked Immunosorbent Assay.

### 4.5. Cyanobacteria against Huntington’s Disease

Huntington’s disease (HD) is a genetic ND that follows an autosomal-dominant inheritance pattern, affecting around 10.6–13.7 per 100,000 individuals [125]. It is caused by the expansion of a cytosine–adenine–guanine (GAG) trinucleotide repeat in the huntingtin (HTT) gene, resulting in a mutant huntingtin protein (mHTT) with an abnormally long polyQ tract. This mutation is fully penetrant at 40 or more repeats [126]. The propensity of these proteins and polyQ N-terminal fragments to aggregate results in the formation of intranuclear inclusions, which are characteristic of HD. mHTT transcripts are also toxic through a gain-of-function mechanism. This disrupts cellular functions, with compromised proteostasis, mitochondrial dysfunction, aberrant immune activation, synaptic excitotoxicity, neuroinflammation, oxidative stress, and defective transcription. The consequence is neuronal death, particularly in the striatum, where medium-spiny neurons (MSNs) are the most vulnerable [9]. HD presents as a triad of motor, cognitive, and emotional impairments. The signature clinical feature is chorea, characterized by involuntary and uncontrolled movements, but it also encompasses a broad range of neuropsychiatric disturbances, including mood disorders and dementia. It typically manifests in adulthood between the ages of 30 and 50, progressing relentlessly with significant disability and is ultimately fatal, with an average survival of 18 years [125,126].

There is currently no cure for HD and available treatments are limited to symptomatic management, such as tetrabenazine and deutetrabenazine to reduce chorea. Disease-modifying therapies are being investigated, including DNA/gene therapies, RNA modulation, stem cell-based therapies, and immunization against mHTT [9,127].

There is limited evidence suggesting the potential of cyanobacteria-derived products in treating HD. The primary mechanism of action is through reducing neurotoxicity and oxidative stress caused by polyQ aggregation (Table 5).

The anti-proteostasis potential of phycocyanin, isolated from *Leptolyngbya* sp. N62D, was demonstrated in *C. elegans* AM141, a model of HD polyQ tract expansion [30]. It expresses polyQ fused to a yellow reporter protein (polyQ::YFP) in muscle cells and becomes progressively paralyzed with age, mimicking the disease [128]. The treatment with phycocyanin (100 μg/mL) in the medium, both in the presence and absence of paraquat, which is a potent oxidative stress inducer, led to a significant decrease in the formation of polyQ::YFP aggregates by 0.63-fold and 0.53-fold, respectively. The treatment also markedly increased the survival rate of AM141 worms, whether paraquat was present or not. Furthermore, phycocyanin (100 μg/mL) demonstrated anti-aging activity in wild-type *C. elegans* (N2) by increasing the mean lifespan, the pharyngeal pumping, and the locomotion rate. It also showed antioxidant potential in vitro, with radical scavenging and reducing power abilities, as well as in vivo, by enhancing tolerance to oxidative stress and thermotolerance of *C. elegans* [30].

Zhong et al. (2021) [129] studied the geroprotective effects of polysaccharides derived from *Nostoc sphaeroides* colonies in *C. elegans* HA759, another model of HD. This transgenic strain exhibits human polyQ expansions in ASH neurons, replicating the HD phenotype and displays impaired avoidance behavior. Exposure to oligosaccharides (NOS-HCA and NOS-TFA) chemically derived from *N. sphaeroides* polysaccharides, at a concentration of 0.5 mg/mL, improved the chemosensory avoidance index of worms, protecting them from polyQ-mediated neurotoxicity. They also upregulated genes linked to stress and proteostasis, namely the glutathione S-transferase gene (gst-4), the catalase gene (ctl-2), and the heat shock protein genes (hsp-6 and hsp-60). In addition, both polysaccharides and their derived oligosaccharides possessed in vitro antioxidant activity, as they scavenged ABTS and DPPH free radicals (2 mg/mL), and in vivo (0.5 mg/mL), by increasing the survival rate of *C. elegans* under both oxidative stress and normal condition [129].

**Table 5:** Cyanobacteria-derived products with neuroprotective activity against HD.

Strain	Compound/Extract	Effect	In Vitro Assays	In Vivo Assays	Reference
*Leptolyngbya* sp. N62DM	Phycocyanin	Anti-polyQ aggregation. Antioxidant. Increased lifespan.	DPPH assay. FRAP assay. SRSA assay. R-Power assay.	N2 *Caenorhabditis elegans*: Life span assay. Pharyngeal pumping and locomotion assays. DCFH-DA fluorescence staining. Stress resistance assay. DAF-16::GFP localization *Caenorhabditis elegans* AM141: PolyQ aggregation assay. Paraquat sensitivity assay. Life span assay. DAF-16::GFP localization	[30]
*Nostoc sphaeroides*	Chemically derived oligosaccharides (NOS-HCA, NOS-TFA)	Improved chemosensory behavior. Improved lifespan. Antioxidant. Gene modulation.	ABTS assay. DPPH assay.	N2 *Caenorhabditis elegans*: Oxidative survival assay. Lifespan assay. qPCR. *Caenorhabditis elegans* HA759: Oxidative survival assay. Chemosensory behavior assay. Lifespan assay. qPCR (gst-4, ctl-2, hsp-6, and hsp-6).	[129]

Abbreviations: polyQ—Polyglutamine. DPPH—2,2-Diphenyl-1-picrylhydrazyl. FRAP—Ferric reducing ability of plasma. SRSA—Superoxide radical scavenging activity. R-Power—Reducing power. DCFH-DA—Dichlorodihydrofluorescein diacetate. DAF-16—Forkhead box protein ortholog gene. GFP—Green fluorescent protein. ABTS—(2,2′-azino-bis(3-ethylbenzothiazoline-6-sulfonic acid)). qPCR—Real-Time Polymerase chain reaction. gst-4—Glutathione S-transferase 4 gene. ctl-2—Peroxisomal catalase 1 gene. hsp-6—Heat shock protein 6 gene. hsp-60—Heat shock protein 60 gene.

## 5. Conclusions

The increasing burden of NDs on aging populations requires urgent attention. Due to limited progress in research, there is a high demand for new therapies. Given the remarkable chemical prolificacy of cyanobacteria and their ability to produce neuroactive compounds, this review aimed to explore the anti-neurodegenerative potential of cyanobacterial natural products.

The data presented show that multiple in silico, in vitro, and in vivo studies support the neuroprotective potential of cyanobacteria. This suggests their ability to combat neurodegeneration through various mechanisms, including acting as enzyme and protein aggregation inhibitors, antioxidants, anti-inflammatories, immunomodulators, or gene modulators. Given that NDs are associated with multiple cellular malfunctions, a multi-target drug strategy such as this, as a standalone treatment or as adjuvant therapy, may prove to be very effective.

The variety of treatment options presented is noteworthy. Several results were credited to complex extracts or whole cyanobacteria, which contain multiple active components that may interact to produce additive/synergistic effects. Moreover, there were also isolated compounds, such as tasiamide B, which highlight the structural and biological diversity of cyanobacteria. The products showcased a range of delivery methods and formulations, with an emphasis on dietary supplements, implying a possible use as nutraceuticals. Furthermore, both regimens of pre-treatment and treatment were investigated, with positive results in both cases, indicating the importance of prevention in NDs.

*Spirulina* was found to be the most versatile among the strains of cyanobacteria mentioned and phycocyanin, which is found in most cyanobacterial strains, was the most studied compound. However, the potential of cyanobacteria in combating NDs is still largely untapped. Further investigating other genera such as *Nostoc* or *Lyngbya* and applying high-throughput screening techniques is worthwhile as each strain has the potential to produce unique sets of compounds that can be valuable. It is also crucial to expand the research to other therapeutic targets and NDs such as ALS, HD, and prion diseases, as there is still a paucity of research on this subject.

Despite the evidence presented and the numerous preclinical studies conducted, translating these insights into clinical applications can be challenging. The assays and animal models may not fully capture the complexity of NDs and pharmacokinetic issues such as the bioavailability, efficacy, and safety of the products can also be a concern. In fact, many cyanobacterial neuroactive compounds are actual neurotoxins. Moreover, the few human clinical trials that have been conducted mostly focused on behavioral/cognitive improvements in patients rather than evaluating molecular markers specific to NDs, suggesting the need for further research in this area.

In conclusion, while cyanobacteria demonstrate promise as a potential treatment option, this field is still in its infancy and further in-depth research is necessary to fully comprehend and harness the potential of cyanobacteria in battling NDs.

## Data Availability

Not applicable.

## References

[1] Wilson D.M., Cookson M.R., Van Den Bosch L., Zetterberg H., Holtzman D.M., Dewachter I. (2023). Hallmarks of neurodegenerative diseases. Cell.

[2] Erkkinen M.G., Kim M.-O., Geschwind M.D. (2018). Clinical Neurology and Epidemiology of the Major Neurodegenerative Diseases. Cold Spring Harb. Perspect. Biol..

[3] Hou Y., Dan X., Babbar M., Wei Y., Hasselbalch S.G., Croteau D.L., Bohr V.A. (2019). Ageing as a risk factor for neurodegenerative disease. Nat. Rev. Neurol..

[4] Ding C., Wu Y., Chen X., Chen Y., Wu Z., Lin Z., Kang D., Fang W., Chen F. (2022). Global, regional, and national burden and attributable risk factors of neurological disorders: The Global Burden of Disease study 1990–2019. Front. Public Health.

[5] Kumar D., Md Ashraf G., Bilgrami A.L., Imtaiyaz Hassan M. (2022). Emerging therapeutic developments in neurodegenerative diseases: A clinical investigation. Drug Discov. Today.

[6] Breijyeh Z., Karaman R. (2020). Comprehensive Review on Alzheimer’s Disease: Causes and Treatment. Molecules.

[7] Gouda N.A., Elkamhawy A., Cho J. (2022). Emerging Therapeutic Strategies for Parkinson’s Disease and Future Prospects: A 2021 Update. Biomedicines.

[8] Masrori P., Van Damme P. (2020). Amyotrophic lateral sclerosis: A clinical review. Eur. J. Neurol..

[9] Tabrizi S.J., Flower M.D., Ross C.A., Wild E.J. (2020). Huntington disease: New insights into molecular pathogenesis and therapeutic opportunities. Nat. Rev. Neurol..

[10] Chen D., Zhang T., Lee T.H. (2020). Cellular Mechanisms of Melatonin: Insight from Neurodegenerative Diseases. Biomolecules.

[11] Porro C., Cianciulli A., Panaro M.A. (2020). The Regulatory Role of IL-10 in Neurodegenerative Diseases. Biomolecules.

[12] Atanasov A.G., Zotchev S.B., Dirsch V.M., Supuran C.T. (2021). Natural products in drug discovery: Advances and opportunities. Nat. Rev. Drug Discov..

[13] Newman D.J., Cragg G.M. (2020). Natural Products as Sources of New Drugs over the Nearly Four Decades from 01/1981 to 09/2019. J. Nat. Prod..

[14] Pentón-Rol G., Marín-Prida J., McCarty M.F. (2021). C-Phycocyanin-derived phycocyanobilin as a potential nutraceutical approach for major neurodegenerative disorders and COVID-19-induced damage to the nervous system. Curr. Neuropharmacol..

[15] Trotta T., Porro C., Cianciulli A., Panaro M.A. (2022). Beneficial effects of spirulina consumption on brain health. Nutrients.

[16] Calella P., Cerullo G., Di Dio M., Liguori F., Di Onofrio V., Gallè F., Liguori G. (2022). Antioxidant, anti-inflammatory and immunomodulatory effects of spirulina in exercise and sport: A systematic review. Front. Nutr..

[17] Waditee-Sirisattha R., Kageyama H., Kageyama H., Waditee-Sirisattha R. (2022). Chapter 1—Cyanobacterial cells. Cyanobacterial Physiology.

[18] Whitton B.A., Potts M., Whitton B.A. (2012). Introduction to the Cyanobacteria. Ecology of Cyanobacteria II: Their Diversity in Space and Time.

[19] Morone J., Alfeus A., Vasconcelos V., Martins R. (2019). Revealing the potential of cyanobacteria in cosmetics and cosmeceuticals—A new bioactive approach. Algal Res..

[20] Tiwari A.K., Tiwari B.S. (2020). Cyanotherapeutics: An emerging field for future drug discovery. Appl. Phycol..

[21] AlFadhly N.K.Z., Alhelfi N., Altemimi A.B., Verma D.K., Cacciola F. (2022). Tendencies Affecting the Growth and Cultivation of Genus Spirulina: An Investigative Review on Current Trends. Plants.

[22] Khalifa S.A.M., Shedid E.S., Saied E.M., Jassbi A.R., Jamebozorgi F.H., Rateb M.E., Du M., Abdel-Daim M.M., Kai G.-Y., Al-Hammady M.A.M. (2021). Cyanobacteria—From the Oceans to the Potential Biotechnological and Biomedical Applications. Mar. Drugs.

[23] Singh R.K., Tiwari S.P., Rai A.K., Mohapatra T.M. (2011). Cyanobacteria: An emerging source for drug discovery. J. Antibiot..

[24] Castaneda A., Ferraz R., Vieira M., Cardoso I., Vasconcelos V., Martins R. (2021). Bridging Cyanobacteria to Neurodegenerative Diseases: A New Potential Source of Bioactive Compounds against Alzheimer’s Disease. Mar. Drugs.

[25] Nugumanova G., Ponomarev E.D., Askarova S., Fasler-Kan E., Barteneva N.S. (2023). Freshwater Cyanobacterial Toxins, Cyanopeptides and Neurodegenerative Diseases. Toxins.

[26] Calabrese G., Molzahn C., Mayor T. (2022). Protein interaction networks in neurodegenerative diseases: From physiological function to aggregation. J. Biol. Chem..

[27] Sweeney P., Park H., Baumann M., Dunlop J., Frydman J., Kopito R., McCampbell A., Leblanc G., Venkateswaran A., Nurmi A. (2017). Protein misfolding in neurodegenerative diseases: Implications and strategies. Transl. Neurodegener..

[28] Peng C., Trojanowski J.Q., Lee V.M.Y. (2020). Protein transmission in neurodegenerative disease. Nat. Rev. Neurol..

[29] Nuzzo D., Presti G., Picone P., Galizzi G., Gulotta E., Giuliano S., Mannino C., Gambino V., Scoglio S., Di Carlo M. (2018). Effects of the Aphanizomenon flos-aquae Extract (Klamin^®^) on a Neurodegeneration Cellular Model. Oxidative Med. Cell. Longev..

[30] Singh N.K., Sonani R.R., Awasthi A., Prasad B., Patel A.R., Kumar J., Madamwar D. (2016). Phycocyanin moderates aging and proteotoxicity in *Caenorhabditis elegans*. J. Appl. Phycol..

[31] Singh A., Kukreti R., Saso L., Kukreti S. (2019). Oxidative Stress: A Key Modulator in Neurodegenerative Diseases. Molecules.

[32] Olufunmilayo E.O., Gerke-Duncan M.B., Holsinger R.M.D. (2023). Oxidative Stress and Antioxidants in Neurodegenerative Disorders. Antioxidants.

[33] Ghanbari A., Vafaei A.A., Naghibi nasab F.S., Attarmoghaddam M., Bandegi A.R., Moradi- Kor N. (2019). Spirulina microalgae improves memory deficit induced by scopolamine in male pup rats: Role of oxidative stress. S. Afr. J. Bot..

[34] Moradi-Kor N., Ghanbari A., Rashidipour H., Bandegi A.R., Yousefi B., Barati M., Kokhaei P., Rashidy-Pour A. (2020). Therapeutic Effects of *Spirulina platensis* Against Adolescent Stress-Induced Oxidative Stress, Brain-Derived Neurotrophic Factor Alterations and Morphological Remodeling in the Amygdala of Adult Female Rats. J. Exp. Pharmacol..

[35] Bermejo-Bescós P., Piñero-Estrada E., Villar del Fresno Á.M. (2008). Neuroprotection by *Spirulina platensis* protean extract and phycocyanin against iron-induced toxicity in SH-SY5Y neuroblastoma cells. Toxicol. Vitr..

[36] Pérez-Juárez A., Chamorro G., Alva-Sánchez C., Paniagua-Castro N., Pacheco-Rosado J. (2016). Neuroprotective effect of Arthrospira (Spirulina) platensis against kainic acid-neuronal death. Pharm. Biol..

[37] Marín-Prida J., Pentón-Rol G., Rodrigues F.P., Alberici L.C., Stringhetta K., Leopoldino A.M., Naal Z., Polizello A.C.M., Llópiz-Arzuaga A., Rosa M.N. (2012). C-Phycocyanin protects SH-SY5Y cells from oxidative injury, rat retina from transient ischemia and rat brain mitochondria from Ca^2+^/phosphate-induced impairment. Brain Res. Bull..

[38] Kwon H.S., Koh S.-H. (2020). Neuroinflammation in neurodegenerative disorders: The roles of microglia and astrocytes. Transl. Neurodegener..

[39] Rauf A., Badoni H., Abu-Izneid T., Olatunde A., Rahman M.M., Painuli S., Semwal P., Wilairatana P., Mubarak M.S. (2022). Neuroinflammatory Markers: Key Indicators in the Pathology of Neurodegenerative Diseases. Molecules.

[40] Chen J.-C., Liu K.S., Yang T.-J., Hwang J.-H., Chan Y.-C., Lee I.T. (2012). Spirulina and C-phycocyanin reduce cytotoxicity and inflammation-related genes expression of microglial cells. Nutr. Neurosci..

[41] Bigagli E., D’Ambrosio M., Cinci L., Pieraccini G., Romoli R., Biondi N., Niccolai A., Rodolfi L., Tredici M.R., Luceri C. (2023). A comparative study of metabolites profiles, anti-inflammatory and antioxidant activity of methanolic extracts from three Arthrospira strains in RAW 264.7 macrophages. Algal Res..

[42] Chei S., Oh H.-J., Song J.-H., Seo Y.-J., Lee K., Kim K.-J., Lee B.-Y. (2020). Spirulina maxima extract prevents activation of the NLRP3 inflammasome by inhibiting ERK signaling. Sci. Rep..

[43] Piovan A., Battaglia J., Filippini R., Dalla Costa V., Facci L., Argentini C., Pagetta A., Giusti P., Zusso M. (2021). Pre- and Early Post-treatment With *Arthrospira platensis* (Spirulina) Extract Impedes Lipopolysaccharide-triggered Neuroinflammation in Microglia. Front. Pharmacol..

[44] Khalil S.R., Khalifa H.A., Abdel-Motal S.M., Mohammed H.H., Elewa Y.H.A., Mahmoud H.A. (2018). *Spirulina platensis* attenuates the associated neurobehavioral and inflammatory response impairments in rats exposed to lead acetate. Ecotoxicol. Environ. Saf..

[45] Armada-Moreira A., Gomes J.I., Pina C.C., Savchak O.K., Gonçalves-Ribeiro J., Rei N., Pinto S., Morais T.P., Martins R.S., Ribeiro F.F. (2020). Going the Extra (Synaptic) Mile: Excitotoxicity as the Road Toward Neurodegenerative Diseases. Front. Cell. Neurosci..

[46] Lee H.Y., Ryu G.H., Choi W.Y., Yang W.S., Lee H.W., Ma C.J. (2018). Protective effect of water extracted Spirulina maxima on glutamate-induced neuronal cell death in mouse hippocampal HT22 cell. Pharmacogn. Mag..

[47] Procházková T., Sychrová E., Javůrková B., Večerková J., Kohoutek J., Lepšová-Skácelová O., Bláha L., Hilscherová K. (2017). Phytoestrogens and sterols in waters with cyanobacterial blooms—Analytical methods and estrogenic potencies. Chemosphere.

[48] Tan J.W., Kim M.K. (2016). Neuroprotective Effects of Biochanin A against β-Amyloid-Induced Neurotoxicity in PC12 Cells via a Mitochondrial-Dependent Apoptosis Pathway. Molecules.

[49] LePage K.T., Goeger D., Yokokawa F., Asano T., Shioiri T., Gerwick W.H., Murray T.F. (2005). The neurotoxic lipopeptide kalkitoxin interacts with voltage-sensitive sodium channels in cerebellar granule neurons. Toxicol. Lett..

[50] Livingston G., Huntley J., Sommerlad A., Ames D., Ballard C., Banerjee S., Brayne C., Burns A., Cohen-Mansfield J., Cooper C. (2020). Dementia prevention, intervention, and care: 2020 report of the Lancet Commission. Lancet.

[51] (2023). 2023 Alzheimer’s disease facts and figures. Alzheimer’s Dement..

[52] Doroszkiewicz J., Mroczko B. (2022). New possibilities in the therapeutic approach to Alzheimer’s Disease. Int. J. Mol. Sci..

[53] Yu T.-W., Lane H.-Y., Lin C.-H. (2021). Novel therapeutic approaches for Alzheimer’s disease: An updated review. Int. J. Mol. Sci..

[54] Gholami A., Minai-Tehrani D., Eriksson L.A. (2023). In silico and in vitro studies confirm Ondansetron as a novel acetylcholinesterase and butyrylcholinesterase inhibitor. Sci. Rep..

[55] Mahmood N.A., Carmichael W.W. (1987). Anatoxin-a (s), an anticholinesterase from the cyanobacterium Anabaena flos-aquae NRC-525-17. Toxicon.

[56] Becher P.G., Baumann H.I., Gademann K., Jüttner F. (2009). The cyanobacterial alkaloid nostocarboline: An inhibitor of acetylcholinesterase and trypsin. J. Appl. Phycol..

[57] Becher P.G., Beuchat J., Gademann K., Jüttner F. (2005). Nostocarboline: Isolation and synthesis of a new cholinesterase inhibitor from Nostoc 78-12A. J. Nat. Prod..

[58] Fiore M.F., de Lima S.T., Carmichael W.W., McKinnie S.M.K., Chekan J.R., Moore B.S. (2020). Guanitoxin, re-naming a cyanobacterial organophosphate toxin. Harmful Algae.

[59] Rodgers K.J., Main B.J., Samardzic K. (2018). Cyanobacterial Neurotoxins: Their Occurrence and Mechanisms of Toxicity. Neurotox. Res..

[60] Fagundes M.B., Alvarez-Rivera G., Mendiola J.A., Bueno M., Sánchez-Martínez J.D., Wagner R., Jacob-Lopes E., Zepka L.Q., Ibañez E., Cifuentes A. (2021). Phytosterol-rich compressed fluids extracts from *Phormidium autumnale* cyanobacteria with neuroprotective potential. Algal Res..

[61] Refaay D.A., Abdel-Hamid M.I., Alyamani A.A., Abdel Mougib M., Ahmed D.M., Negm A., Mowafy A.M., Ibrahim A.A., Mahmoud R.M. (2022). Growth Optimization and Secondary Metabolites Evaluation of *Anabaena variabilis* for Acetylcholinesterase Inhibition Activity. Plants.

[62] Touliabah H.E., Refaay D.A. (2023). Enhancement of Anticancer, Antibacterial, and Acetylcholinesterase Inhibition Activities from *Oscillatoria sancta* under Starvation Conditions. Water.

[63] Khemiri S., Khelifi N., Messaoud C., Smaali I. (2023). Bioprospecting of microalgae for a potential use as enzyme inhibitors, anti-ageing and prebiotic agents. Biocatal. Agric. Biotechnol..

[64] Hampel H., Hardy J., Blennow K., Chen C., Perry G., Kim S.H., Villemagne V.L., Aisen P., Vendruscolo M., Iwatsubo T. (2021). The Amyloid-β Pathway in Alzheimer’s Disease. Mol. Psychiatry.

[65] Luo Y.-C., Jing P. (2020). Molecular Interaction of Protein-Pigment C-Phycocyanin with Bovine Serum Albumin in a Gomphosis Structure Inhibiting Amyloid Formation. Int. J. Mol. Sci..

[66] Liu Y., Jovcevski B., Pukala T.L. (2019). C-Phycocyanin from Spirulina Inhibits α-Synuclein and Amyloid-β Fibril Formation but Not Amorphous Aggregation. J. Nat. Prod..

[67] Liu J., Chen W., Xu Y., Ren S., Zhang W., Li Y. (2015). Design, synthesis and biological evaluation of tasiamide B derivatives as BACE1 inhibitors. Bioorganic Med. Chem..

[68] Liu Y., Zhang W., Li L., Salvador L.A., Chen T., Chen W., Felsenstein K.M., Ladd T.B., Price A.R., Golde T.E. (2012). Cyanobacterial Peptides as a Prototype for the Design of Potent β-Secretase Inhibitors and the Development of Selective Chemical Probes for Other Aspartic Proteases. J. Med. Chem..

[69] Al-Awadhi F.H., Ratnayake R., Paul V.J., Luesch H. (2016). Tasiamide F, a potent inhibitor of cathepsins D and E from a marine cyanobacterium. Bioorganic Med. Chem..

[70] Singh K.N., Hasan S.S., Kumar J., Raj I., Pathan A.A., Parmar A., Shakil S., Gourinath S., Madamwar D. (2014). Crystal Structure and Interaction of Phycocyanin with β-Secretase: A Putative Therapy for Alzheimer’s Disease. CNS Neurol. Disord. Drug Targets.

[71] Chaubey M.G., Patel S.N., Rastogi R.P., Srivastava P.L., Singh A.K., Madamwar D., Singh N.K. (2019). Therapeutic potential of cyanobacterial pigment protein phycoerythrin: In silico and in vitro study of BACE1 interaction and in vivo Aβ reduction. Int. J. Biol. Macromol..

[72] Koh E.-J., Kim K.-J., Song J.-H., Choi J., Lee H.Y., Kang D.-H., Heo H.J., Lee B.-Y. (2017). Spirulina maxima Extract Ameliorates Learning and Memory Impairments via Inhibiting GSK-3β Phosphorylation Induced by Intracerebroventricular Injection of Amyloid-β 1–42 in Mice. Int. J. Mol. Sci..

[73] Galizzi G., Deidda I., Amato A., Calvi P., Terzo S., Caruana L., Scoglio S., Mulè F., Di Carlo M. (2023). Aphanizomenon flos-aquae (AFA) Extract Prevents Neurodegeneration in the HFD Mouse Model by Modulating Astrocytes and Microglia Activation. Int. J. Mol. Sci..

[74] Klamin. https://www.klamathshop.eu/klamin/.

[75] AphaMax. https://www.klamathshop.eu/aphamax/.

[76] Chandrasekaran V., Hediyal T.A., Anand N., Kendaganna P.H., Gorantla V.R., Mahalakshmi A.M., Ghanekar R.K., Yang J., Sakharkar M.K., Chidambaram S.B. (2023). Polyphenols, Autophagy and Neurodegenerative Diseases: A Review. Biomolecules.

[77] Cortés-Gómez M.-Á., Llorens-Álvarez E., Alom J., del Ser T., Avila J., Sáez-Valero J., García-Ayllón M.-S. (2021). Tau phosphorylation by glycogen synthase kinase 3β modulates enzyme acetylcholinesterase expression. J. Neurochem..

[78] Elsonbaty S.M., Ismail A.F.M. (2020). Nicotine encourages oxidative stress and impairment of rats’ brain mitigated by *Spirulina platensis* lipopolysaccharides and low-dose ionizing radiation. Arch. Biochem. Biophys..

[79] Zhou T., Liu Y., Wang Q., Dou Q., Li X., Pan Y., Meng L., Xue T. (2021). *Spirulina platensis* alleviates high fat diet-induced cognitive impairment in mice via the gut-brain axis. J. Funct. Foods.

[80] Chen L., Liu Y.-c., Tan H., Zhang Y., Xu J., Liu W.-l., Li Z.-y., Li W.-p. (2019). Santacruzamate A Ameliorates AD-Like Pathology by Enhancing ER Stress Tolerance through Regulating the Functions of KDELR and Mia40-ALR in vivo and in vitro. Front. Cell. Neurosci..

[81] Malm T., Koistinaho J., Kanninen K. (2011). Utilization of APPswe/PS1dE9 Transgenic Mice in Research of Alzheimer’s Disease: Focus on Gene Therapy and Cell-Based Therapy Applications. Int. J. Alzheimer’s Dis..

[82] Yousef M.I., Abdou H.M., Abd Elkader H.-T.A.E.A., Hussein H.K., Abou Samra W.E.M. (2020). Neuroprotective Potential of *Spirulina Platensis* against Aluminium Chloride-Induced Neural Degeneration. Curr. Top. Nutraceutical Res..

[83] Abdelghany A.K., Gamal A., Abdel-Wahab A., Abdel-Razik A.-R.H., El-Samannoudy S.I., Ibrahim M.A., Hassan W.H., El-Ela F.I.A. (2023). Evaluating the neuroprotective effect of *Spirulina platensis*–loaded niosomes against Alzheimer’s disease induced in rats. Drug Deliv. Transl. Res..

[84] Cammann D., Lu Y., Cummings M.J., Zhang M.L., Cue J.M., Do J., Ebersole J., Chen X., Oh E.C., Cummings J.L. (2023). Genetic correlations between Alzheimer’s disease and gut microbiome genera. Sci. Rep..

[85] Imai Y., Koseki Y., Hirano M., Nakamura S. (2021). Nutrigenomic Studies on the Ameliorative Effect of Enzyme-Digested Phycocyanin in Alzheimer’s Disease Model Mice. Nutrients.

[86] Li Z., Gan L., Yan S., Yan Y., Huang W. (2020). Effect of C-phycocyanin on HDAC3 and miRNA-335 in Alzheimer’s disease. Transl. Neurosci..

[87] Agrawal M., Perumal Y., Bansal S., Arora S., Chopra K. (2020). Phycocyanin alleviates ICV-STZ induced cognitive and molecular deficits via PI3-Kinase dependent pathway. Food Chem. Toxicol..

[88] Choi W.-Y., Lee W.-K., Kim T.-H., Ryu Y.-K., Park A., Lee Y.-J., Heo S.-J., Oh C., Chung Y.-C., Kang D.-H. (2022). The Effects of Spirulina maxima Extract on Memory Improvement in Those with Mild Cognitive Impairment: A Randomized, Double-Blind, Placebo-Controlled Clinical Trial. Nutrients.

[89] Tamtaji O.R., Heidari-soureshjani R., Asemi Z., Kouchaki E. (2023). The effects of spirulina intake on clinical and metabolic parameters in Alzheimer’s disease: A randomized, double-blind, controlled trial. Phytother. Res..

[90] Bloem B.R., Okun M.S., Klein C. (2021). Parkinson’s disease. Lancet.

[91] Balestrino R., Schapira A.H.V. (2020). Parkinson disease. Eur. J. Neurol..

[92] Zaman V., Shields D.C., Shams R., Drasites K.P., Matzelle D., Haque A., Banik N.L. (2021). Cellular and molecular pathophysiology in the progression of Parkinson’s disease. Metab. Brain Dis..

[93] Macedo D., Bertolin T.E., Oro T., Backes L.T.H., Brás I.C., Santos C.N., Tenreiro S., Outeiro T.F. (2017). Phycocyanin protects against Alpha-Synuclein toxicity in yeast. J. Funct. Foods.

[94] Latif S., Jahangeer M., Maknoon Razia D., Ashiq M., Ghaffar A., Akram M., El Allam A., Bouyahya A., Garipova L., Ali Shariati M. (2021). Dopamine in Parkinson’s disease. Clin. Chim. Acta.

[95] Chamorro G., Pérez-Albiter M., Serrano-García N., Mares-Sámano J.J., Rojas P. (2006). Spirulina maxima pretreatment partially protects against 1-methyl-4-phenyl-1,2,3,6-tetrahydropyridine neurotoxicity. Nutr. Neurosci..

[96] Tobón-Velasco J.C., Palafox-Sánchez V., Mendieta L., García E., Santamaría A., Chamorro-Cevallos G., Limón I.D. (2013). Antioxidant effect of Spirulina (*Arthrospira*) maxima in a neurotoxic model caused by 6-OHDA in the rat striatum. J. Neural Transm..

[97] Chattopadhyaya I., Gupta S., Mohammed A., Mushtaq N., Chauhan S., Ghosh S. (2015). Neuroprotective effect of Spirulina fusiform and amantadine in the 6-OHDA induced Parkinsonism in rats. BMC Complement. Altern. Med..

[98] Xu F.-h., Qiu Y.-z., Zhang Y., Yang F.-h., Ji M.-m., Liu K.-c., Jin M., Zhang S.-s., Li B. (2023). The molecular mechanism of three novel peptides from C-phycocyanin alleviates MPTP-induced Parkinson’s disease-like pathology in zebrafish. Food Funct..

[99] Aryal B., Lee Y. (2019). Disease model organism for Parkinson disease: Drosophila melanogaster. BMB Rep..

[100] Salim M., Subandi M., Yuniarti Y. Neuroprotective Benefits of S. platensis Extract on Drosophila melanogaster Model of Parkinson’s Disease. Proceedings of the 1st International Conference on Islam, Science and Technology, ICONISTECH 2019.

[101] Kumar A., Christian P.K., Panchal K., Guruprasad B.R., Tiwari A.K. (2017). Supplementation of spirulina (*Arthrospira platensis*) improves lifespan and locomotor activity in paraquat-sensitive DJ-1β Δ93 flies, a Parkinson’s Disease model in Drosophila melanogaster. J. Diet. Suppl..

[102] Gopinath A., Mackie P., Hashimi B., Buchanan A.M., Smith A.R., Bouchard R., Shaw G., Badov M., Saadatpour L., Gittis A. (2022). DAT and TH expression marks human Parkinson’s disease in peripheral immune cells. NPJ Park. Dis..

[103] Zhang F., Lu J., Zhang J.-g., Xie J.-x. (2015). Protective effects of a polysaccharide from *Spirulina platensis* on dopaminergic neurons in an MPTP-induced Parkinson’s disease model in C57BL/6J mice. Neural Regen. Res..

[104] Lopes M.J.P., Delmondes G.d.A., Leite G.M.d.L., Cavalcante D.R.A., Aquino P.É.A.d., Lima F.A.V.d., Neves K.R.T., Costa A.S., Oliveira H.D.d., Bezerra Felipe C.F. (2022). The protein-rich fraction from *Spirulina platensis* exerts neuroprotection in hemiparkinsonian rats by decreasing brain inflammatory-related enzymes and glial fibrillary acidic protein expressions. J. Med. Food.

[105] Lima F.A.V., Joventino I.P., Joventino F.P., de Almeida A.C., Neves K.R.T., do Carmo M.R., Leal L.K.A.M., de Andrade G.M., de Barros Viana G.S. (2017). Neuroprotective activities of *Spirulina platensis* in the 6-OHDA model of Parkinson’s disease are related to its anti-inflammatory effects. Neurochem. Res..

[106] Pabon M.M., Jernberg J.N., Morganti J., Contreras J., Hudson C.E., Klein R.L., Bickford P.C. (2012). A spirulina-enhanced diet provides neuroprotection in an α-synuclein model of Parkinson’s disease. PLoS ONE.

[107] Strömberg I., Gemma C., Vila J., Bickford P.C. (2005). Blueberry-and spirulina-enriched diets enhance striatal dopamine recovery and induce a rapid, transient microglia activation after injury of the rat nigrostriatal dopamine system. Exp. Neurol..

[108] Yang J.H., Rempe T., Whitmire N., Dunn-Pirio A., Graves J.S. (2022). Therapeutic Advances in Multiple Sclerosis. Front. Neurol..

[109] Dobson R., Giovannoni G. (2019). Multiple sclerosis—A review. Eur. J. Neurol..

[110] Filippi M., Bar-Or A., Piehl F., Preziosa P., Solari A., Vukusic S., Rocca M.A. (2018). Multiple sclerosis. Nat. Rev. Dis. Primers.

[111] Procaccini C., De Rosa V., Pucino V., Formisano L., Matarese G. (2015). Animal models of Multiple Sclerosis. Eur. J. Pharmacol..

[112] Pentón-Rol G., Martínez-Sánchez G., Cervantes-Llanos M., Lagumersindez-Denis N., Acosta-Medina E.F., Falcón-Cama V., Alonso-Ramírez R., Valenzuela-Silva C., Rodríguez-Jiménez E., Llópiz-Arzuaga A. (2011). C-Phycocyanin ameliorates experimental autoimmune encephalomyelitis and induces regulatory T cells. Int. Immunopharmacol..

[113] Cervantes-Llanos M., Lagumersindez-Denis N., Marín-Prida J., Pavón-Fuentes N., Falcon-Cama V., Piniella-Matamoros B., Camacho-Rodríguez H., Fernández-Massó J.R., Valenzuela-Silva C., Raíces-Cruz I. (2018). Beneficial effects of oral administration of C-Phycocyanin and Phycocyanobilin in rodent models of experimental autoimmune encephalomyelitis. Life Sci..

[114] Pentón-Rol G., Lagumersindez-Denis N., Muzio L., Bergami A., Furlan R., Fernández-Massó J.R., Nazabal-Galvez M., Llópiz-Arzuaga A., Herrera-Rolo T., Veliz-Rodriguez T. (2016). Comparative neuroregenerative effects of C-phycocyanin and IFN-beta in a model of multiple sclerosis in mice. J. Neuroimmune Pharmacol..

[115] Gardón D.P., Cervantes-Llanos M., Matamoros B.P., Rodríguez H.C., Tan C.-y., Marín–Prida J., Falcón-Cama V., Pavón-Fuentes N., Lemus J.G., Ruiz L.d.l.C.B. (2022). Positive effects of Phycocyanobilin on gene expression in glutamate-induced excitotoxicity in SH-SY5Y cells and animal models of multiple sclerosis and cerebral ischemia. Heliyon.

[116] Marín-Prida J., Pavón-Fuentes N., Lagumersindez-Denis N., Camacho-Rodríguez H., García-Soca A.M., Sarduy-Chávez R.d.l.C., Vieira É.L.M., Carvalho-Tavares J., Falcón-Cama V., Fernández-Massó J.R. (2022). Anti-inflammatory mechanisms and pharmacological actions of phycocyanobilin in a mouse model of experimental autoimmune encephalomyelitis: A therapeutic promise for multiple sclerosis. Front. Immunol..

[117] Longinetti E., Fang F. (2019). Epidemiology of amyotrophic lateral sclerosis: An update of recent literature. Curr. Opin. Neurol..

[118] Goutman S.A., Hardiman O., Al-Chalabi A., Chió A., Savelieff M.G., Kiernan M.C., Feldman E.L. (2022). Emerging insights into the complex genetics and pathophysiology of amyotrophic lateral sclerosis. Lancet Neurol..

[119] Mead R.J., Shan N., Reiser H.J., Marshall F., Shaw P.J. (2023). Amyotrophic lateral sclerosis: A neurodegenerative disorder poised for successful therapeutic translation. Nat. Rev. Drug Discov..

[120] Johnson S.A., Fang T., De Marchi F., Neel D., Van Weehaeghe D., Berry J.D., Paganoni S. (2022). Pharmacotherapy for Amyotrophic Lateral Sclerosis: A Review of Approved and Upcoming Agents. Drugs.

[121] De Paola M., Mariani A., Bigini P., Peviani M., Ferrara G., Molteni M., Gemma S., Veglianese P., Castellaneta V., Boldrin V. (2012). Neuroprotective effects of toll-like receptor 4 antagonism in spinal cord cultures and in a mouse model of motor neuron degeneration. Mol. Med..

[122] Krishnaraj R.N., Kumari S.S.S., Mukhopadhyay S.S. (2016). Antagonistic molecular interactions of photosynthetic pigments with molecular disease targets: A new approach to treat AD and ALS. J. Recept. Signal Transduct..

[123] Bonifacino T., Zerbo R.A., Balbi M., Torazza C., Frumento G., Fedele E., Bonanno G., Milanese M. (2021). Nearly 30 Years of Animal Models to Study Amyotrophic Lateral Sclerosis: A Historical Overview and Future Perspectives. Int. J. Mol. Sci..

[124] Garbuzova-Davis S., C Bickford P. (2010). Neuroprotective effect of Spirulina in a mouse model of ALS. Open Tissue Eng. Regen. Med. J..

[125] McColgan P., Tabrizi S.J. (2018). Huntington’s disease: A clinical review. Eur. J. Neurol..

[126] Bates G.P., Dorsey R., Gusella J.F., Hayden M.R., Kay C., Leavitt B.R., Nance M., Ross C.A., Scahill R.I., Wetzel R. (2015). Huntington disease. Nat. Rev. Dis. Primers.

[127] Pan L., Feigin A. (2021). Huntington’s Disease: New Frontiers in Therapeutics. Curr. Neurol. Neurosci. Rep..

[128] Nedosekin D.A., Chen T., Ayyadevara S., Zharov V.P., Shmookler Reis R.J. (2021). Label-free photothermal disruption of cytotoxic aggregates rescues pathology in a C. elegans model of Huntington’s disease. Sci. Rep..

[129] Zhong G., Pan W., Huang Z., Guo K., Hu J., Liu P., Chen S., Wang Y., Ai L., Huang Z. (2021). Physicochemical and geroprotective comparison of Nostoc sphaeroides polysaccharides across colony growth stages and with derived oligosaccharides. J. Appl. Phycol..

